# Cancer Relevance of Circulating Antibodies Against LINE-1 Antigens in Humans

**DOI:** 10.1158/2767-9764.CRC-23-0289

**Published:** 2023-11-08

**Authors:** Alexandra V. Vylegzhanina, Ivan A. Bespalov, Ksenia A. Novototskaya-Vlasova, Brandon M. Hall, Anatoli S. Gleiberman, Han Yu, Olga V. Leontieva, Katerina I. Leonova, Oleg V. Kurnasov, Andrei L. Osterman, Grace K. Dy, Alexey A. Komissarov, Elena Vasilieva, Jeff Gehlhausen, Akiko Iwasaki, Christine B. Ambrosone, Takemasa Tsuji, Junko Matsuzaki, Kunle Odunsi, Ekaterina L. Andrianova, Andrei V. Gudkov

**Affiliations:** 1 Genome Protection, Inc., Buffalo, New York.; 2 Roswell Park Comprehensive Cancer Center, Buffalo, New York.; 3 Sanford Burnham Prebys Medical Discovery Institute, La Jolla, California.; 4 I.V. Davydovsky Clinical City Hospital, Moscow, Russia.; 5 A.I. Yevdokimov Moscow State University of Medicine and Dentistry, Moscow, Russia.; 6 Yale University, New Haven, Connecticut.; 7 Howard Hughes Medical Institute, Chevy Chase, Maryland.; 8 University of Chicago Medicine Comprehensive Cancer Center, Chicago, Illinois.

## Abstract

Long interspersed nuclear element-1 (LINE-1 or L1), the most abundant family of autonomous retrotransposons occupying over 17% of human DNA, is epigenetically silenced in normal tissues by the mechanisms involving p53 but is frequently derepressed in cancer, suggesting that L1-encoded proteins may act as tumor-associated antigens recognized by the immune system. In this study, we established an immunoassay to detect circulating autoantibodies against L1 proteins in human blood. Using this assay in >2,800 individuals with or without cancer, we observed significantly higher IgG titers against L1-encoded ORF1p and ORF2p in patients with lung, pancreatic, ovarian, esophageal, and liver cancers than in healthy individuals. Remarkably, elevated levels of anti–ORF1p-reactive IgG were observed in patients with cancer with disease stages 1 and 2, indicating that the immune response to L1 antigens can occur in the early phases of carcinogenesis. We concluded that the antibody response against L1 antigens could contribute to the diagnosis and determination of immunoreactivity of tumors among cancer types that frequently escape early detection.

**Significance:**

The discovery of autoantibodies against antigens encoded by L1 retrotransposons in patients with five poorly curable cancer types has potential implications for the detection of an ongoing carcinogenic process and tumor immunoreactivity.

## Introduction

Long interspersed nuclear element-1 (L1) is a non-long terminal repeat (non-LTR) retrotransposon comprising hundreds of thousands of copies accumulated during mammalian evolution (1). In humans, approximately half a million copies of L1 collectively occupy over 17% of the DNA (2). Most of these sequences are functionally deficient because of truncations, internal rearrangements, and mutations. Only approximately 150 copies of L1 elements are retrotransposition competent in humans (3). Among these full-length L1s, only a few “hot” loci contribute to bulk L1 mRNA expression (4). Intact L1 sequences are approximately 6 kb long and encode two polypeptides, ORF1p and ORF2p (5). ORF1p is an RNA-binding protein (6) and ORF2p is a multifunctional protein that combines reverse transcriptase (RT) and endonuclease (integrase) activities, which are essential for replication and expansion of L1 and other non-autonomous retrotransposons (7). The L1 replication machinery is believed to be responsible for most reverse transcription–driven integration events in the genome, including those involved in the expansion of short interspersed nuclear elements (SINE), pericentromeric satellite DNA, and processed pseudogenes (1).

L1 elements are transcriptionally silenced in most normal cells owing to multiple mechanisms of epigenetic repression, including DNA methylation and chromatin modifications; the latter mechanism involves histone deacetylases [e.g., Sirtuin 6 (8)], tumor suppressors p53 (9), and retinoblastoma protein (10). L1 desilencing creates multiple deleterious risks for cells and organisms (11). In germline cells, it can lead to inherited diseases due to insertional mutagenesis (12). In somatic cells, it drives multiple mechanisms of genetic and epigenetic instability stemming from insertional mutagenesis (13, 14), DNA damage caused by L1 endonuclease activity (15), and activation of IFN- and NFκB-mediated inflammatory responses (16, 17).

L1 elements are frequently derepressed in human tumors, a large proportion of which express L1 antigens (11, 18) and experience L1 expansion in their genome (15, 19). L1 derepression contributing to cancer genome instability through insertional mutagenesis (9, 10, 11, 13) and DNA damage caused by L1 endonuclease (20). Moreover, activation of L1 can promote tumor treatment resistance by activation of prosurvival inflammatory pathways independent of retrotranspositions (21).

L1 activity differs between and within cancer types and may change during cancer progression (22). The most recent data define esophageal adenocarcinoma and lung squamous cell carcinoma as having the highest rates of retrotransposition among human cancers. In a study that analyzed 246 pancreatic cancer samples, approximately half exhibited an active retrotransposition process (13). L1 ORF1p and ORF2p proteins have been considered as cancer biomarkers (23–25). According to IHC staining for ORF1p, 47% of 1,027 samples representing more than 20 cancer types were positive for L1 expression (26). ORF2p was detected less frequently in tumors, which is not surprising given the many-fold lower expression of ORF2p compared with ORF1p (27). Within ORF2p-positive tumors, a switch from cytoplasmic to nuclear staining for ORF2p been reported during tumor progression (28).

A substantial effort toward developing computational techniques for analyzing L1 sequences in genomic DNA and in liquid biopsies has resulted in detection of frequent acquisition of new copies of L1 in tumors (13, 29) and demonstrated a significant decrease in L1 methylation in cell-free DNA of patients with cancer as compared with healthy controls (30).

However, neither of these observations has been translated into clinically feasible diagnostic assays suitable for early cancer detection.

A similar situation has developed around the detection of L1 proteins in circulation. For a long-time attempt to detect ORF1p and ORF2p in the blood of patients with cancer had been unsuccessful (18). It was only recently that Taylor and colleagues reported the development of an ultrasensitive technique enabling detection of femtomolar levels ORF1p, which demonstrated its presence in the blood of patients with multiple cancer types, especially frequently in the subjects with ovarian and colorectal cancers (31).

During normal development, L1 expression is limited to the early stages of embryogenesis (32), brain neurons, especially in elderly subjects (33, 34), and specific cell subpopulations in the testis (35). Because these sites are all behind immunologic barriers and, therefore, do not participate in the formation of immune tolerance to autoantigens, one would expect that L1 antigens expressed in tumors should be recognized by the immune system as tumor-associated antigens and induce an adaptive immune response. On the basis of this assumption, we hypothesized that the presence of L1-positive cancer is associated with the development of an autoantibody response to L1 antigens. A major possibility for such a response is supported by a report of anti-ORF1p antibodies in patients with autoimmune diseases (36). We expected that antibodies to L1 proteins could be an easier detectable biomarker of L1 derepression than L1 antigens themselves because it results from an amplified response of adaptive immunity to the presence of antigens that have limited access to circulation. In addition to translational applications, such a biomarker could provide new insights in tumor immunoreactivity and the mechanisms of interaction of L1 retrotransposon with the host immune system.

On the basis of these considerations, we developed an immunoassay capable of specific semiquantitative detection of circulating antibodies against L1 antigens in human sera. Using this immunoassay, we analyzed >2,500 blood samples from patients with 14 cancer types along with >300 cancer-free individuals, either healthy or with cancer-unrelated health problems. The obtained results support our hypothesis, demonstrating a frequent association between the ongoing carcinogenic process and elevated levels of anti-ORF1p antibodies in blood, which can reach unprecedented titers. Remarkably, this association was observed in both the early and advanced disease stages, supporting the potential utility of anti-ORF1p antibodies for cancer detection and determination of tumor immunoreactivity.

## Materials and Methods

### Serum Sample Collections

All procedures involving human samples were performed in accordance with the ethical standards of the Institutional Review Boards (IRB) and with the 1964 Helsinki Declaration and its later amendments or comparable ethical standards that included obtaining written informed consent and conducted under IRB-approved protocols.

Serum samples from patients with cancer were obtained from the Roswell Park Data Bank and BioRepository (DBBR), a CCSG Shared Resource which prospectively consents new patients to provide blood samples, to complete an epidemiologic risk factor questionnaire, and for linkage of samples to clinical data and to tumor tissue samples. Newly diagnosed patients are consented prior to cancer treatment to investigators with IRB-approved protocols for collection of blood samples, completion of a risk factor questionnaire, and permission to link their samples to tumor tissue and to clinical data. DBBR specimens are rigorously collected, processed, and stored so that the integrity of potential analytes will be consistent across all patients and controls. Blood is drawn into tubes that are immediately sent to the DBBR laboratory for standardized preparation of serum, plasma, red blood cells, buffy coat, and DNA; per SOP, aliquots are frozen within 1 hour of collection and stored in liquid nitrogen to minimize sample degradation. Samples were collected between 2003 and 2021, patients with cancer were included if they were a newly diagnosed patient with a blood sample drawn and banked in the DBBR or Ovarian Bank with information in the Cancer Registry.

In addition to enrolling patients with cancer, the DBBR also uses a number of approaches to enroll men and women with no personal history of cancer. These include controls recruited at community events, visitors accompanying patients with cancer and employee volunteers. Samples are collected, processed, and stored using the same protocols as those used for patients with cancer.

The sample set used in this study consists of 2,815 well-annotated samples from the DBBR and Ovarian Bank of RPCCC (female *N* = 1,663; male *N* = 1,152) with the age range varying between 18 and 95 years old. It includes 14 cancer categories classified according the organ of cancer origin: 24 samples from glioblastoma brain tumor; 24 samples from breast cancer (infiltrating ductal carcinoma and infiltrating lobular carcinoma); 907 samples of lung cancer (non–small cell carcinoma and small cell carcinoma), 24 samples from colorectal adenocarcinoma, 24 samples of soft-tissue sarcoma, 377 samples from esophageal adenocarcinoma and squamous cell carcinoma, 24 samples of renal cell carcinoma; 217 samples of hepatocellular carcinoma; 979 samples of serous ovarian carcinoma, 124 samples of pancreatic adenocarcinoma; 24 samples of prostatic adenocarcinoma; 23 samples of skin malignant melanoma, 20 samples from gastric adenocarcinoma, 24 samples of urinary bladder cancer (papillary urothelial carcinoma and transitional cell carcinoma).

We also included 78 samples from DBBR controls and 200 from healthy volunteers obtained from Innovation Research (https://www.innov-research.com/), as well as samples from I.V. Davydovsky Clinical City Hospital (Moscow Department of Healthcare, Moscow, Russia collected September 2021–April 2022. The study was approved by the Moscow City Ethics Committee of the Research Institute of the Organization of Health and Healthcare Management (protocol #LABS022021) and performed according to the Helsinki Declaration. These included samples from healthy volunteers (*N* = 74), patients with non-cancer with recent myocardial infarction (MI; *N* = 37) and patients with pulmonary diseases, chronic obstructive pulmonary disease and/or pneumonia (COPD/PN; *N* = 33). A total non-cancer dataset consists of 352 serum samples; among them: female *N* = 194, male *N* = 158 with the age ranging between 20 and 89 years old.

Systemic lupus erythematosus (SLE) patient samples were utilized from a repository generated in a previous study (37) under a protocol approved by the Institutional Review Committee of Yale University (New Haven, CT; HIC #0303025105). All enrolled patients with SLE were diagnosed according to American College of Rheumatology 1997 criteria. Plasma samples were obtained after processing peripheral blood from sodium-heparin–coated tubes and stored as aliquots at –80°C.

### Recombinant L1 Antigens

The protein-coding DNA sequence corresponding to human L1 ORF1p described in Uniport Entry Q9UN81 (LORF1_HUMAN) was custom synthesized with codon optimization for expression in *Escherichia coli* (GenScript Biotech; see the protocol for L1 ORF1p expression and purification in Supplementary Data).

The protein-coding DNA sequence corresponding to fragment 367-771 of human L1 ORF2p described in Uniport Entry O00370 (LORF2_HUMAN); or as human NAG13 protein (GenBank ID: AAG27485.1) was custom synthesized with codon optimization for expression in *E. coli* (GenScript Biotech). Cloning and expression of 367-771 fragment (413 aa; 47.6 kDa) of human L1 ORF2p was performed according to the same protocol as described above for human ORF1p, with exception of using the size exclusion chromatography (SEC) as final purification step after immobilized metal ion affinity chromatography (IMAC) purification/refolding. HiLoad 16/600 Superdex-200 FPLC column (GE Healthcare) was equilibrated with 50 mmol/L Tris-HCl, pH 8.5; 2 mol/L Urea; 0.5 mol/L NaCl; 0.15% Brij35; 2.5 mmol/L DTT. SEC-eluted samples were subject to buffer exchange on Fast-Desalt FPLC column HiPrep 26/10 (GE Healthcare) in 50 mmol/L Tris-HCl, pH 8.0; 0.3 mol/L NaCl; 0.5 mmol/L DTT; 0.05% Tween80; 5% Glycerol, flash frozen and stored at −80°С.

### ORF1p-derived Peptides

Seventeen overlapping 25-mer ORF1p-derived peptides (Supplementary Table S1) containing cysteine were custom synthesized at GenScript and consequently conjugated with BSA (Sigma) through Sulfo-SMCC (Thermo Fisher Scientific) according to protocol at fishersci.com “SMCC and Sulfo-SMCC User Guide” (Pub. No. MAN0011295 C.0).

### Anti-ORF1p and Anti-ORF2p Immunoassays

The anti-ORF1p and anti-ORF2p assays are designed to detect anti-ORF1p or anti-ORF2p antibodies using an indirect ELISA method performed in plates coated with ORF1p or a fragment of ORF2p proteins encoded by an L1 retroelement capable of expression in humans. The titers of anti–ORF1p-reactive IgG antibodies in human serum were measured in assay plates coated with a full-length ORF1p protein as antigen; the titers of anti–ORF2p-reactive IgG antibodies in human serum were measured in assay plates coated with a RT domain (amino acids residues 367-771) as antigen (see the immunoassays protocol in Supplementary Data).

### Anti-p53 and Anti-ORF1p Peptide Antibody ELISA

Recombinant Human Tumor Protein p53 (RayBiotech, Code 230-00639-10) was diluted to 1 µg/mL in a 2 mol/L urea/PBS coating buffer; or each of 17 ORF1p 25-mer peptides conjugated with BSA were diluted to 2 µg/mL in PBS coating buffer and added to Black MaxiSorp 96-well assay plates (50 µL/well). The protocols for antibody detection for p53 and ORF1p peptides are provided in Supplementary Data.

### Sandwich ELISA for Detection Human ORF1p and ORF2p

#### Capture Antibodies

Affinity purified rabbit anti-L1 [anti-ORF1p or anti-ORF2p (RT fragment)] polyclonal antibodies were ordered from GenScript (Custom Polyclonal Antibody Production Service). Rabbits were immunized for 7 weeks with human ORF1p protein or human ORF2p peptides. Sera were collected and purified after final immunization using antigen affinity chromatography for ORF1p and protein A affinity chromatography for ORF2p.

#### Detection Antibodies

Anti-ORF1p mouse polyclonal sera were obtained in our laboratory by conventional immunization procedure with human recombinant ORF1p protein; anti-ORF2p rat affinity purified polyclonal antibodies were obtained from ProSci AbServices after rats’ immunization with human ORF2p protein (RT fragment).

#### Sandwich ELISA

A total of 50 µL of affinity purified rabbit anti-hORF1p or anti-ORF2p polyclonal antibody immobilized on Black MaxiSorp 96-well assay overnight at 4°C. After washing and blocking with Assay Buffer, the plates were incubated sequentially with serum samples and detection antibodies: anti-ORF1p mouse or anti-ORF2p rat Abs (1:500 in casein assay buffer, 50 µL/well). After washing, goat anti-mouse IgG+IgM+IgA (heavy and light chain) or anti-rat IgG horseradish peroxidase–conjugate in dilution 1:1,000 in Assay buffer (50 µL/well) was added and incubated for 1 hour at room temperature, following washing and addition of QuantaBlu fluorogenic peroxidase substrate (Thermo Fisher Scientific) for 15 minutes at room temperature. Fluorescence was measured at an excitation wavelength of 320 nm and an emission wavelength of 420 nm using a plate reader (Tecan Infinite M1000 Pro).

### Cells with Inducible Expression of L1

The inducible L1 reporter plasmid used to generate HeLa tet-L1/GLucAI cells (pBH001) was generated through a series of successive PCR-based cloning steps performed by GenScript. pBH001 is comprised of a tetracycline-regulated bidirectional promoter for inducible expression of both firefly luciferase [a fragment cloned from the pTRE3G-BI-Luc control plasmid (Takara Bio)] and a recoded human L1 sequence (encoding both ORF1 and ORF2) cloned from the L1-neo-TET plasmid (Addgene # 51284; a gift from Astrid Roy-Engel; ref. 38). A custom antisense intron-interrupted *Gaussia* Luciferase (GLuc)-based retrotransposition readout cassette (GLucAI; driven by SV40 promoter) synthesized by GenScript was inserted within the 3′-untranslated region of L1 in the antisense orientation. Elements originating from the PB-gRNA-puro plasmid (Addgene #121121; a gift from Pablo Navarro; ref. 39) were used as a backbone for the construct, and includes PiggyBac transposase-specific short inverted terminal repeats (40) that flank the sequence described above and includes a puromycin resistance gene for selection of positive PiggyBac-mediated integration of the cassette into recipient cells.

To generate a stable population of HeLa cells carrying an integrated tetL1-GLucAI construct, HeLa TetOn3G cells (Takara) were cotransfected with the tet-L1/GLucAI donor plasmid (pBH201) and the Super PiggyBac Transposase Expression Vector (System Biosciences) at a 5:1 plasmid ratio, as recommended by the manufacturer's protocol. Transfections were performed using LipoD293 reagent (SignaGen) with a total of 3 µg plasmid DNA per 10-cm dish, following the manufacturer's protocol. A total of 72 hours after transfection, cells were selected for 3 days in 1 µg/mL puromycin. After two passages, an additional 72 hours selection was performed to ensure acquisition of a population with stably integration of the tetL1-GLucAI cassette. Clones were isolated via limiting dilution into 96-well plates, and subsequently characterized for doxycycline-stimulated acquisition of *Gaussia* luciferase activity. Clone 9A8 was used in this study.

### Retrotransposition Assay

HeLa tet-L1/GLucAI were plated at 10^6^ cells per 10-cm plate and expression of the integrated tet-L1/GLucAI cassette was induced by addition of doxycycline to a final concentration of 400 ng/mL in the culture medium. After 48 hours, GLuc activity in the conditioned medium (indicative of successful L1-mediated retrotransposition activity) was measured using a microplate luminometer immediately following addition of an equal volume of 2x GLuc reagent [50 µmol/L coelenterazine (GoldBio) in D-PBS containing 300 mmol/L sodium ascorbate (Sigma) and 0.2% Triton X-100 (Sigma)]. HeLa tet-L1/GLucAI cells without induction were used as control and prepared by the same way (Supplementary Fig. S1).

### Western Immunoblotting

To assess specificity of the detected antibodies to L1 proteins, we analyzed serum samples from patients with cancer that had 10^7^–10^8^ anti-ORF1p IgG titers in the anti-ORF1p assay as the source of antibodies for immunofluorescent staining and immunoblot analysis of HeLa cells expressing tetracycline-inducible human L1 (see the protocol in Supplementary Data).

### Immunofluorescence Staining of Cells

Cells were fixed in 4% formaldehyde in PBS for 15 minutes at 4°C, washed 3x in PBS, kept at 4°C for 1 hour in PBS for staining, blocked in blocking solution (5% normal donkey serum, 0.2% triton x-100, PBS or TBS, 1% glycine) for 15 minutes. Cells were incubated with primary antibody (mouse monoclonal from Millipore, MABS1152 dilution 1:400 in block solution or human anti-ORF1p antibody (#11, #766, #899, #865 1:50,000 dilution or rabbit anti-human ORF1p custom made 1:200 dilution for 30–60 minutes, washed 6x in PBS, cells were incubated with secondary antibody (donkey anti-mouse, anti-human or anti-rabbit Jackson ImmunoResearch laboratories Inc) Cy3 or AlexaFluor488 conjugated, 2 µg/mL for 30 minutes, washed 6x in PBS. Nuclei were counterstained with DAPI. Samples were washed 4 hours with several changes of PBS, cleared, and mounted with ProLong Gold antifade reagent. Images were collected with Zeiss AxioImager 2 fluorescent microscope equipped with AxioCam 702 digital camera using ZEN software 2.6 version.

### Statistical Analysis

Statistical analysis and graph plotting were performed using GraphPad Prizm software (Version 9), Microsoft Office Excel and R 4.0.5. To determine the normality of the distribution the Shapiro–Wilk normality test was used. The differences in multiple groups were analyzed by Dunn multiple comparison test with the multiplicity adjusted *P* value. The differences between the two groups were analyzed by the Mann–Whitney *U* test. For correlation analysis Spearman *r* correlation analysis was performed. Logistic regression was performed with disease status as response and anti-ORF1p IgG as independent variable, before and after adjustment for age. Because of the presence of multiple cancer types, the associations of individual cancer types with anti-ORF1p IgG were assessed using linear regression models with log_10_ transformed titer being the outcome while adjusting for age. The Dunnett tests for contrasts of all cancer types with healthy control were utilized for the correction of multiple comparisons. Age was dichotomized at the median (62 years) in regression analyses for robustness considerations. A value of *P* < 0.05 was considered statistically significant in all analyses.

### Data Access Statement

The data generated in this study are included in the article and its Supplementary Figures. Raw data are available upon request without restriction from the corresponding authors.

## Results

### Anti-L1 Antibodies in Patients with a Variety of Cancer Types and in Healthy Individuals

**FIGURE 1:**
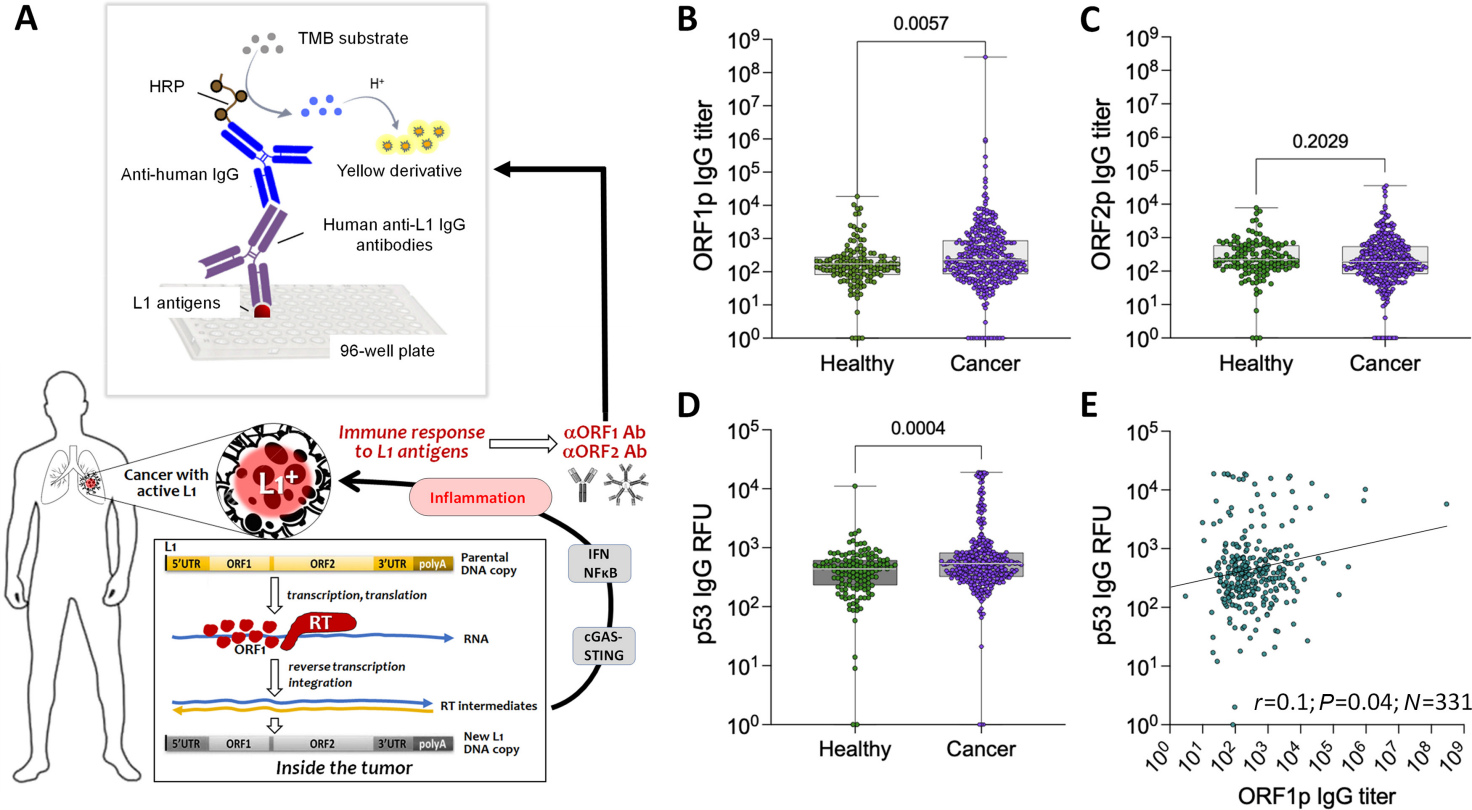
Approach and proof of concept. **A,** Hypotheses: proteins encoded by L1 are expected to be recognized as tumor-associated antigens and become targets for antibody response. This response is stimulated by the cGAS-STING-mediated induction of inflammation. Circulating Abs to L1 antigens can be used as cancer biomarkers if detected by anti-ORF1p and anti-ORF2p immunoassays. Comparison of antibody levels in healthy individuals (*N* = 137) and patients with cancer (*N* = 331) in the assays for anti-ORF1p (**B**), anti-ORF2p (**C**), and p53 (**D**). The significance of differences is assessed by Mann–Whitney *U* test. **E,** Spearman correlation between anti-ORF1p IgG titers and anti-p53 IgG signals in serum samples of patients with cancer (*N* = 331). All *P* value less than 0.05 is considered significant.

The hypotheses underlying this study are schematically illustrated in Fig. 1A. To detect anti-ORF1p or anti-ORF2p antibodies in serum samples, we used an indirect ELISA technique using 96-well plates coated with recombinant polypeptides representing L1 antigens: a full-length ORF1p or a 47-kDa fragment of ORF2p covering the region spanning 367-771 amino acids (see Materials and Methods). We used the full-length human L1 ORF1p sequence with no modifications, because, according to a BLAST search, ORF1p has no significant similarities with other human proteins that could result in cross-reactivity of anti-ORF1p antibodies. The ELISA protocol was optimized to increase the sensitivity and reduce the background (see Materials and Methods) by substituting BSA for casein in the buffer resulting in a 20-fold background reduction.

To test for the existence of the principal phenomenon (that is the development of an antibody response to L1 antigens in patients with cancer), we first analyzed a mixed set of 331 serum samples representing 14 solid tumors, including: ovarian (*N* = 24), breast (*N* = 24), lung (*N* = 24), colorectal (*N* = 24), esophageal (*N* = 24), renal (*N* = 24), liver (*N* = 24), pancreatic (*N* = 24), prostate (*N* = 24), gastric (*N* = 20), bladder (*N* = 24) cancers, soft-tissue sarcoma (*N* = 24), melanoma (*N* = 23), and glioblastoma (*N* = 24), along with 137 healthy individuals. The titers of anti-ORF1p IgG antibodies were significantly higher in the cancer patient population than in healthy subjects [median titer, 213 (interquartile range, IQR, 85–884) vs. 149 [(IQR, 81–280); *P* = 0.0057] (Fig. 1B). The small sample sizes for each cancer type in this study limited our ability to reliably classify which cancer types exhibit elevated anti-L1 antibodies (Supplementary Fig. S2A).

Evaluation of anti-ORF2p IgG titers from the same samples did not show a significant difference between cancer patients and healthy individuals [median titer, 185 (IQR, 82–549) vs. 221 (IQR, 127–590); *P* = 0.2029] (Fig. 1C; Supplementary Fig. S2B). Furthermore, maximal anti-ORF2p IgG titers (35,867) were more than 3 orders of magnitude lower than the maximal titers of anti-ORF1p antibodies (293,944,693).

The same serum samples were also used to detect IgG against p53, a tumor suppressor protein most frequently mutated in cancer, for which autoantibodies have been considered as a biomarker for cancer detection (41). The rationale for comparing humoral immune responses to L1 antigens and p53 is based on the involvement of wild-type p53 in epigenetic repression of L1 (42) and the fact that p53 mutation correlated with L1 ORF1p expression in some cancer types (43). Similar to the antibody response to ORF1p, the same patients with cancer showed a significantly higher elevation in anti-p53 versus healthy subjects [median, 514 (IQR, 321–826) vs. 437 (IQR, 232–614); *P* = 0.0004] (Fig. 1D; Supplementary Fig. S2C). A weak but significant positive correlation was observed between anti-ORF1p and anti-p53 IgG signals (Spearman correlation: *r* = 0.1, *P* = 0.04; Fig. 1E).

**FIGURE 2:**
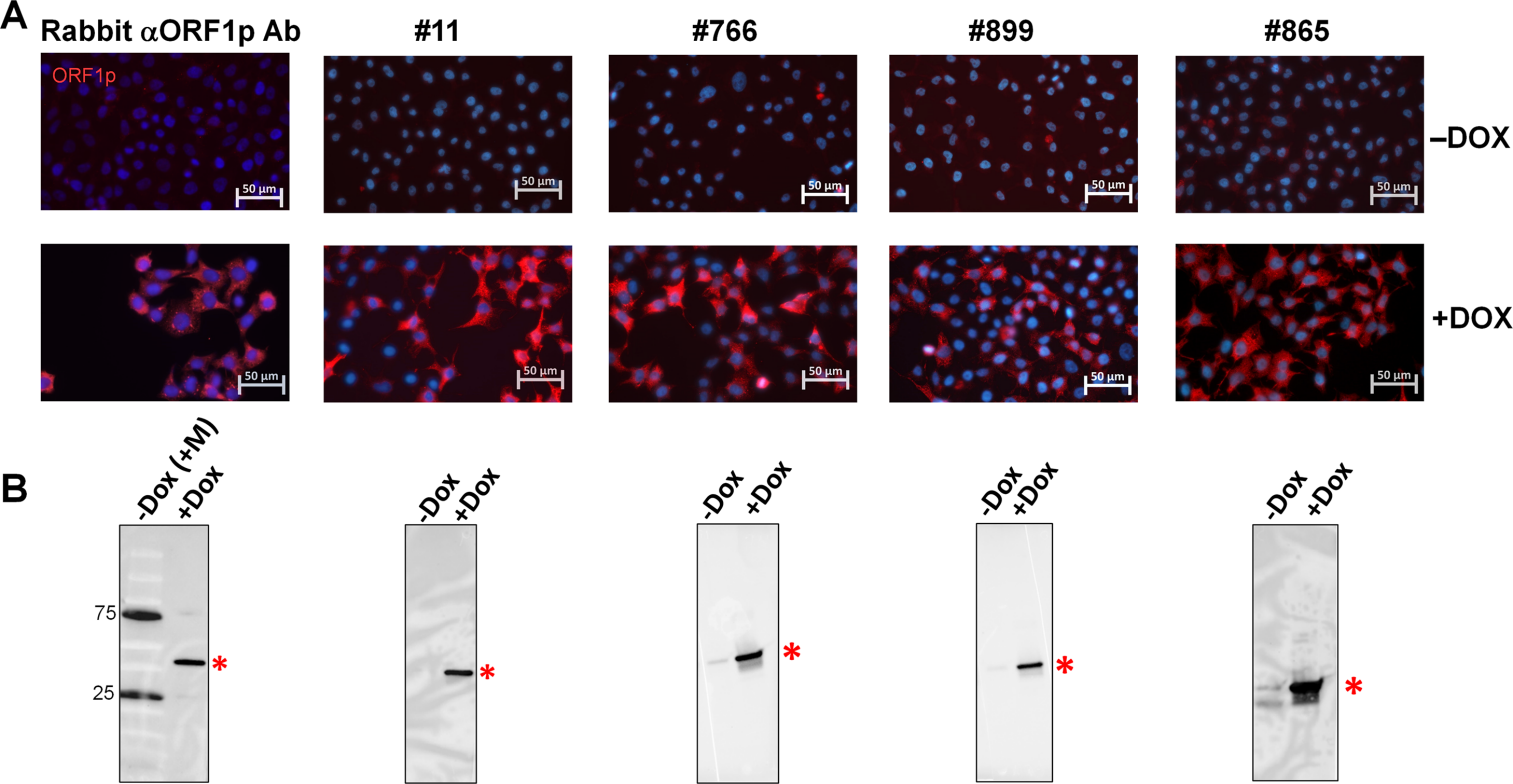
Relevance of antibodies detected by immunoassay to L1 ORF1p. The results of immunofluorescent staining (**A**) and immunoblot analysis (**B**) of HeLa cells transduced with Tet-inducible L1/Gluc reporter construct with (+DOX) and without (−DOX) L1 induction by doxycycline (Supplementary Fig. S1). “Rabbit αORF1p Ab” – polyclonal anti-ORF1p rabbit antiserum. The other panels represent staining with 50,000-fold diluted serum samples from patients with ovarian cancer with 10^7^–10^8^ anti-ORF1p IgG titers in anti-ORF1p immunoassay. “M” stands for the protein size marker added to the sample along with HeLa cells lysate without DOX L1 induction. The asterisks on the bottom panel correspond to ORF1p standard (40-kDa) band.

To assess the specificity of the detected antibodies to L1 proteins, we analyzed serum samples from 4 patients with ovarian cancer with 10^7^–10^8^ anti-ORF1p IgG titers in our immunoassay as the source of antibodies for immunofluorescent staining and immunoblot analysis of HeLa cells expressing tetracycline-inducible human L1 (see Materials and Methods; Supplementary Fig. S1). The analysis revealed bright immunofluorescent staining of HeLa cells with induced L1 expression (but not the same cells without L1 induction; Fig. 2A) and a band with the expected size for ORF1p (40-kDa) on the immunoblots of the lysates of HeLa cells after doxycycline-induced L1 expression (Fig. 2B). These results were similar to those obtained with the control rabbit antibodies against ORF1p (Fig. 2A and B, left). Serum samples with titers below 880 failed to produce signals in both immunofluorescent staining and immunoblots (Supplementary Table S2).

Among the serum samples analyzed, a clear relevance of anti-ORF1p IgG titers in immunoassay with sample positivity in immunofluorescent staining of cells with induced L1 and immunoblot-based detection of ORF1p was observed (Supplementary Table S2; Supplementary Fig. S3). Rare inconsistencies, where anti–ORF1p-positive serum sample failed to detect ORF1p signal in HeLa cells by immunostaining, could be explained either by the specificity of antibodies in these samples to conformational ORF1p epitopes or sensitivity of immunoblot-based detection of ORF1p by low-titers antibodies.

The fact that patients’ serum samples used in dilutions between 1:1,000 and 1:50,000 for immunofluorescence and immunoblot staining demonstrated highly specific recognition of ORF1p among numerous protein present in fixed human cells or human cell lysates indicates that anti-ORF1p IgG appeared as major autoimmune-reactive antibodies in circulation in most subjects with 10^4^–10^9^ anti-ORF1p IgG titers (Fig. 2B; Supplementary Fig. S3).

To map immunoreactive portions within ORF1p polypeptides and more accurately assess specificity of the detected anti-ORF1p IgG, we tested 36 selected serum samples with a range of anti-ORF1p antibody titers using a library of 17 synthetic 26-amino acid long overlapping ORF1p-derived peptides conjugated with BSA (Supplementary Table S1). As evident from the data presented in Supplementary Table S3, samples that had high titers of antibodies to the whole ORF1p were also positive in the ORF1p-derived peptide-based assay. In one case (lung cancer patient #1410), anti-ORF1p-positive serum sample failed to detect any of anti-ORF1p peptides, what could be explained by the same reasons given above for immunoblot-based detection. Peptides derived from the first 60 amino acids of ORF1p were found to represent the most immunogenic part of the ORF1p molecule.

Notably, positive serum samples typically identify multiple peptides derived from distinct segments of ORF1p, underscoring the polyclonal nature of the response. Furthermore, the tested subjects exhibited variations in the range of recognized ORF1p-derived peptides, suggesting that the humoral response varies among individuals.

We were unable to detect ORF2p using immunofluorescent or immunoblot staining, even with serum samples that had 10^4^–10^5^ anti-ORF2p titers in immunoassay. This is consistent with the overall lower Ab levels for this L1 protein but may also be associated with conformational ORF2p epitopes.

To test whether antibody response to L1 antigens is associated with the presence of L1 antigens in circulation, we established a sandwich ELISA (see Materials and Methods) capable of detecting 0.1 ng/mL ORF1p and used it to analyze the same set of serum samples from patients with cancer. To check whether the presence of high antibody levels against ORF1p in the circulation could mask the signal in this assay (Supplementary Fig. S4A), we introduced a protein-antibody denaturing step in the protocol that indeed increased the assay sensitivity in the presence of anti-ORF1p antibodies in model experiments (Supplementary Fig. S4B). Only 3% of tested samples possessed detectable concentrations of ORF1p ranging between 0.12 and 5.5 ng/mL (median 0.36 ng/mL; *N* = 331 cancer patient samples and *N* = 72 healthy individuals) and the denaturation step in the sandwich ELISA did not generate any additional signals. Consistently, these 3% samples did not belong to those with the highest anti-ORF1p IgG titers. These observations indicate the advantage of anti-L1 ORF1p IgG versus ORF1p antigen measurement in the blood, at least within the current limit of detection of ORF1p sandwich ELISA.

### Disease Stage Relevance of Anti-ORF1p IgG Titers

**FIGURE 3:**
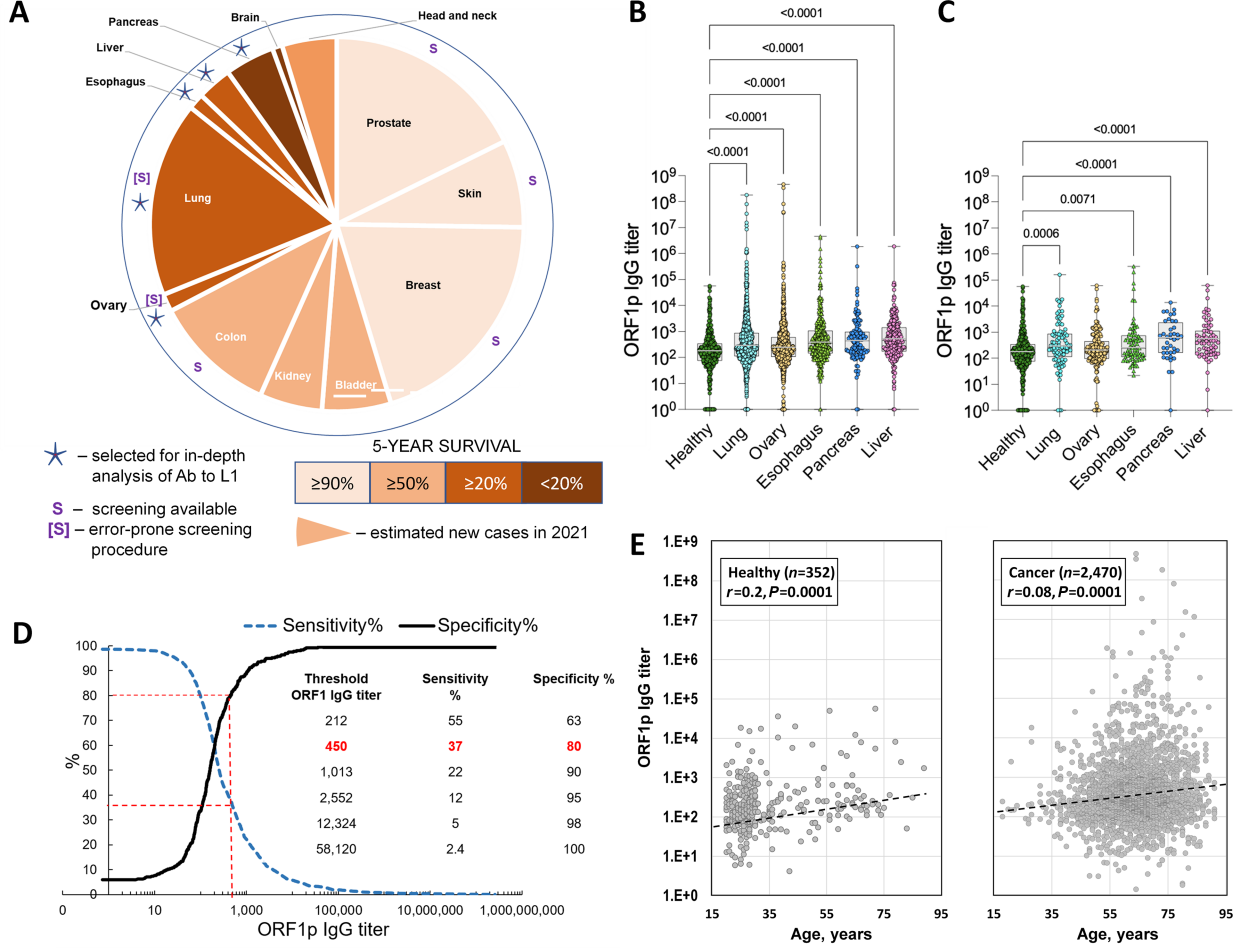
Anti-ORF1p IgG response in serum samples from patients with five cancer types. **A,** Selection of cancers for analysis in anti-ORF1p immunoassay. Sector sizes are proportional to the incidence of these cancers in the United States in 2021 (https://seer.cancer.gov/statfacts/). Color code indicates a 5-year survival. “S” stands for the existence of approved cancer screening protocols. **B,** Detection of anti-ORF1p IgG titers in serum samples of patients with the indicated cancer types of all stages. Sample size: ovary (*N* = 979), esophagus (*N* = 377), lung (*N* = 907), pancreas (*N* = 124), liver (*N* = 217) cancers and healthy (*N* = 352). **C,** The same as panel B; only samples from patients with cancer stages 1 and 2 are shown. Sample size: ovary (*N* = 193), esophagus (*N* = 79), lung (*N* = 90), pancreas (*N* = 40), and liver (*N* = 67) cancers and healthy (*N* = 352). The gray areas in B and C mark samples below the anti-ORF1p IgG titer threshold of 450. Statistics were calculated by Dunn multiple comparison test with adjusted *P* value for anti-ORF1p IgG titers. All *P* value less than 0.05 is considered significant. **D,** Anti-ORF1p immunoassay threshold determination. **E,** Spearman *r* correlation analysis of anti-ORF1p IgG titers and age among healthy individuals (20–89 years of age) or patients with cancer (18–95 years of age).

We expanded our study to several cancer types with poor prognoses and the treatment of which would benefit greatly from early detection. Specifically, we analyzed an additional set of 2,484 serum samples from patients with ovarian (*N* = 979), esophageal (*N* = 377), lung (*N* = 907), pancreatic (*N* = 124), and liver cancers (*N* = 217) which included patients with early (1–2) and advanced (3–4) disease stages (Supplementary Table S4). The rationale for this selection is illustrated in Fig. 3A. The five cancer types selected are characterized by a poor 5-year survival, and four of them (esophageal, pancreatic, liver, and ovarian) currently do not have clinically approved screening protocols in people who are at average risk. The screening procedure currently used for the lung cancer detection in a high-risk population is known to be insufficiently accurate and involves an invasive biopsy collection step (44). These cancers (with the exception of hepatocellular carcinoma) have a high frequency of L1 derepression, as indicated by IHC assessment and the detection of retrotranspositions found in earlier reports (13, 18).

The comparison with 352 samples from healthy individuals revealed significantly elevated anti-ORF1p IgG titers in all five selected cancers as judged by nonparametric Dunn test with adjusted *P* value (Fig. 3B). Healthy [median titer, 160 (IQR, 75–347)] versus lung [248; (IQR, 111–876); *P* < 0.0001], versus ovarian [222 (IQR, 106–607); *P* < 0.0001], versus esophageal [351 (IQR, 143–1,086); *P* < 0.0001], versus pancreatic cancers [436 (IQR, 160–971); *P* < 0.0001], versus hepatocellular carcinoma [453 (IQR, 170–1,443); *P* < 0.0001]. Importantly, these differences remained significant if we limited the analysis to patients with early (1–2) stages of lung (*N* = 90) [median titer, 263 (IQR, 113–874); *P* = 0.0006], esophageal (*N* = 79) [240 (IQR, 111–757); *P* = 0.0071], pancreatic (*N* = 40) [594 (IQR, 162–2,369); *P* < 0.0001] and liver (*N* = 67) [463 (IQR, 155–1,121); *P* < 0.0001] cancers even though the number of cases in each category was substantially smaller (Fig. 3C).

Analysis of anti-ORF2p IgG showed similar, albeit less pronounced, effects. Significantly elevated IgG titers to the ORF2p were found in three out of five selected cancer categories: ovarian [median titer, 442 (IQR, 159–1,378); *P* < 0.0001], pancreatic [352 (IQR, 136–919); *P* < 0.0001], and liver [260 (IQR, 108–748); *P* = 0.0025] cancer versus healthy [167 (IQR, 92–378)] (Supplementary Fig. S5A). As far as the early-stage cancers is concerned, increased anti-ORF2p IgG titers compared with healthy population was found for stages 1–2 of pancreatic [median titer, 697 (IQR, 344–2,102); *P* < 0.0001] and liver [424 (IQR, 185–1,596); *P* < 0.0001] cancers (Supplementary Fig. S5B) opening the opportunity for a more precise early detection of these cancer types if two anti-ORF1p and anti-ORF2p immunoassays are combined. We found a positive correlation between anti-ORF1p and ORF2p IgG titers in all these cancers (Supplementary Fig. S6).

The relationship between the anti-ORF1p IgG titer and cancer risk is visually demonstrated in Fig 3D. While the detection of circulating IgG against L1 antigens is frequent in healthy individuals (∼20% had anti-ORF1p IgG titers exceeding the threshold of 450, yielding an 80% cancer specificity), this parameter cannot be utilized as an indicator of the absence of a carcinogenic process. This observation is further underscored by the ROC curves (Supplementary Fig. S7): the attained AUC values (ranging from 0.6 to 0.7) do not meet the requisite high levels for envisioning the anti-ORF1p immunoassay as a definitive cancer diagnostic tool. Nonetheless, above a specific threshold, the distinction between patients with cancer and cancer-free individuals becomes pronounced, and those with anti-ORF1p IgG titers surpassing 58,120 exhibit nearly negligible prospects of being cancer-free (Fig. 3D).

### Factors Potentially Confounding Cancer Relevance of ORF1p Immunoassay Results

Recent reports on L1 derepression in senescent cells (45) suggest a potential age dependence of the antibody response to L1 antigens. Indeed, we found an increase in anti-ORF1p IgG titers with age (Spearman correlation in healthy: *r* = 0.2, *P* = 0.0001; *N* = 352; 20–89 (mean, 35±16) years of age and cancer: *r* = 0.08; *P* = 0.0001; *N* = 2,470; 18–95 (mean, 64±11) years of age (Fig. 3E)]. The distribution of ages between cancer and healthy subjects is shown in Supplementary Table S5 and graphically in Fig 3E. Univariate logistic regression analysis shows that higher anti-ORF1p IgG titer is significantly associated with increased risk of cancer, with an OR of 1.8 for every 10-fold increase in the titer. We also established a significant (*P* < 0.0001) association between cancer and anti-ORF1p IgG titers after adjusting for age [cancer (*N* = 2,470) and healthy (*N* = 352); 18–95 years of age] (Fig. 3E; Supplementary Table S6), though with an attenuated OR of 1.67. This conclusion held true across all examined cancer types, as indicated in Supplementary Table S7. Moreover, a notable association between cancer and anti-ORF1p IgG titers remained significant even after adjusting for age in the early stages (1 and 2) of esophageal, liver, and pancreatic cancers (Supplementary Table S8). Therefore, although age is correlated with both cancer status and anti-ORF1p IgG titers, we concluded that there is an association between anti-ORF1p IgG titers and cancer that is independent of age.

No racial differences in anti-ORF1p IgG titers were found in healthy or cancer population, as analyzed by Dunn multiple comparison test (Supplementary Table S9). Gender-specific differences were observed only in lung cancer samples, where anti-ORF1p IgG titers were higher in male patients than in female [median titer, 282 (IQR, 127–1,266) vs. 222 (IQR, 100–736); *P* = 0.004; Mann–Whitney *U* test] (Supplementary Table S10). No significant differences were found among the other cancer types analyzed. This intriguing observation might be linked to the documented impacts of tobacco carcinogens on L1 derepression (46, 47) and the higher prevalence of heavy smokers among male individuals with lung cancer (48). However, no dependence of anti-ORF1p IgG titers on smoking history was found neither in patients with lung cancer (Supplementary Fig. S8A), nor in a combined set of pancreatic, esophageal, and liver cancer patients (Supplementary Fig. S8B).

Similarly, we did not find any significant differences in anti-ORF1p IgG titers in a cross-sectional study of patients with cancer treated with radiotherapy, immunotherapy, or chemotherapy compared with non-treated patients with cancer (Supplementary Fig. S9). However, our analysis included only patients whose disease advanced following treatment. Whether anti-ORF1p IgG titers decrease following cancer eradication remains to be addressed.

We also tested the stability of anti-ORF1p IgG levels in a longitudinal study of 18 healthy individuals whose blood samples were collected three times (days 0, 49, and 189) within 6 months. The levels of anti-ORF1p IgG titers ranged substantially between individuals but remained stable within the observation period (Supplementary Fig. S10).

L1 expression can trigger inflammatory pathways mediated by cGAS-STING signaling (45, 49). Therefore, it is important to assess the potential relevance of antibodies against the L1 antigens in inflammation-associated diseases. The titers of IgG against L1 antigens were evaluated via the anti-ORF1p immunoassay in serum samples from patients with recent MI (*N* = 37), as well as from patients with COPD/PN (*N* = 33). We found no statistically significant differences compared with the healthy population, suggesting that COPD/PN and MI do not influence the levels of IgG against L1 antigens (Supplementary Table S11).

Thus, we conclude that anti-ORF1p IgG titer is a cancer-associated parameter that is not confounded by age and gender, race, smoking, inflammation, or cancer treatment history and maintains high stability over time in healthy subjects.

Previous studies reported the presence of anti-ORF1p antibodies in patients with SLE (50). Here we analyzed a set of 17 samples from patients with SLE and found elevated anti-ORF1p (but not anti-ORF2p) IgG titers compared with healthy individuals [median titer, 718 (IQR, 623–1,268) vs. 160 (IQR, 75–347); *P* < 0.0001] (Supplementary Fig. S11A and S11B). These results confirm that the development of an anti-ORF1p IgG response might be common among patients with autoimmune diseases and should be considered during analysis. It is noteworthy that anti-p53 IgG signals in patients with SLE were significantly higher than those in healthy subjects [median signal, 0.073 (IQR, 0.067–0.108) vs. 0.26 (IQR, 0.153–0.498); *P* = 0.011] (Supplementary Fig. S11C).

## Discussion

We developed an immunoassay for the detection of circulating antibodies against L1-encoded proteins and used it for the analysis of >2,800 blood samples that included healthy individuals and patients with a variety of cancers and autoimmune, cardiovascular, and lung diseases. We found that IgG antibodies against ORF1p, and to a lesser extent ORF2p, are frequently detected in humans, indicating that L1 antigens are commonly recognized by the immune system and induce humoral immune responses. Among the disease categories tested, cancer showed the strongest association with elevated serum IgG antibody levels against L1 antigens. Extended testing validated this finding in patients with lung (both non-small cell and small cell lung cancer), esophageal, ovarian, pancreatic, and liver cancers; other cancer types remain to be thoroughly analyzed.

L1 desilencing was previously considered a cancer biomarker detectable by hypomethylation in circulating cell-free DNA in liquid biopsies (18, 51). Here, we demonstrate that epigenetic dysregulation of L1 can generate an additional type of biomarker, circulating autoantibodies against L1 antigens, which can be detected by a blood test. The higher IgG titers observed for ORF1p over ORF2p likely reflect the many-fold higher expression of ORF1p compared with that of ORF2p (23, 27, 32).

At the outset of this study, we postulated that antibodies against L1 antigens in the blood could be more easily detectable than the antigens themselves. This presumption was based on the idea that the immune response amplifies the L1 expression signal. In fact, for a long time, the assays for ORF1p detection have been insufficiently sensitive (18). Only recently, Taylor and colleagues presented an ultrasensitive technique for detecting ORF1p (31), which appears to be a significant advancement in approaching this biomarker. While a systematic comparison of the outcomes of anti-ORF1p antibody and ORF1p protein detection in patients with cancer is pending, the available data already suggest both similarities (such as the strong scoring in ovarian cancer) and potential differences (like the high protein and low antibody test scores in colorectal cancer) between the assays, highlighting their possible complementarity.

Remarkably, elevated levels of anti-L1 IgG antibodies are frequently observed in patients with cancer stages 1 and 2 suggesting that humoral immune response to L1 antigens can occur at early stages of carcinogenic process. In fact, L1 expression frequently occurs in the early stages of malignant transformation and either remains high during tumor progression (11, 52) or, in some instances, decrease (53). This feature distinguishes the anti-L1 antibody response from that of other autoantibodies considered as potential cancer biomarkers, which are limited in their value for early cancer detection because they are more commonly associated with advanced stages of disease [e.g., to p53, heat shock proteins, etc. (54)].

**FIGURE 4:**
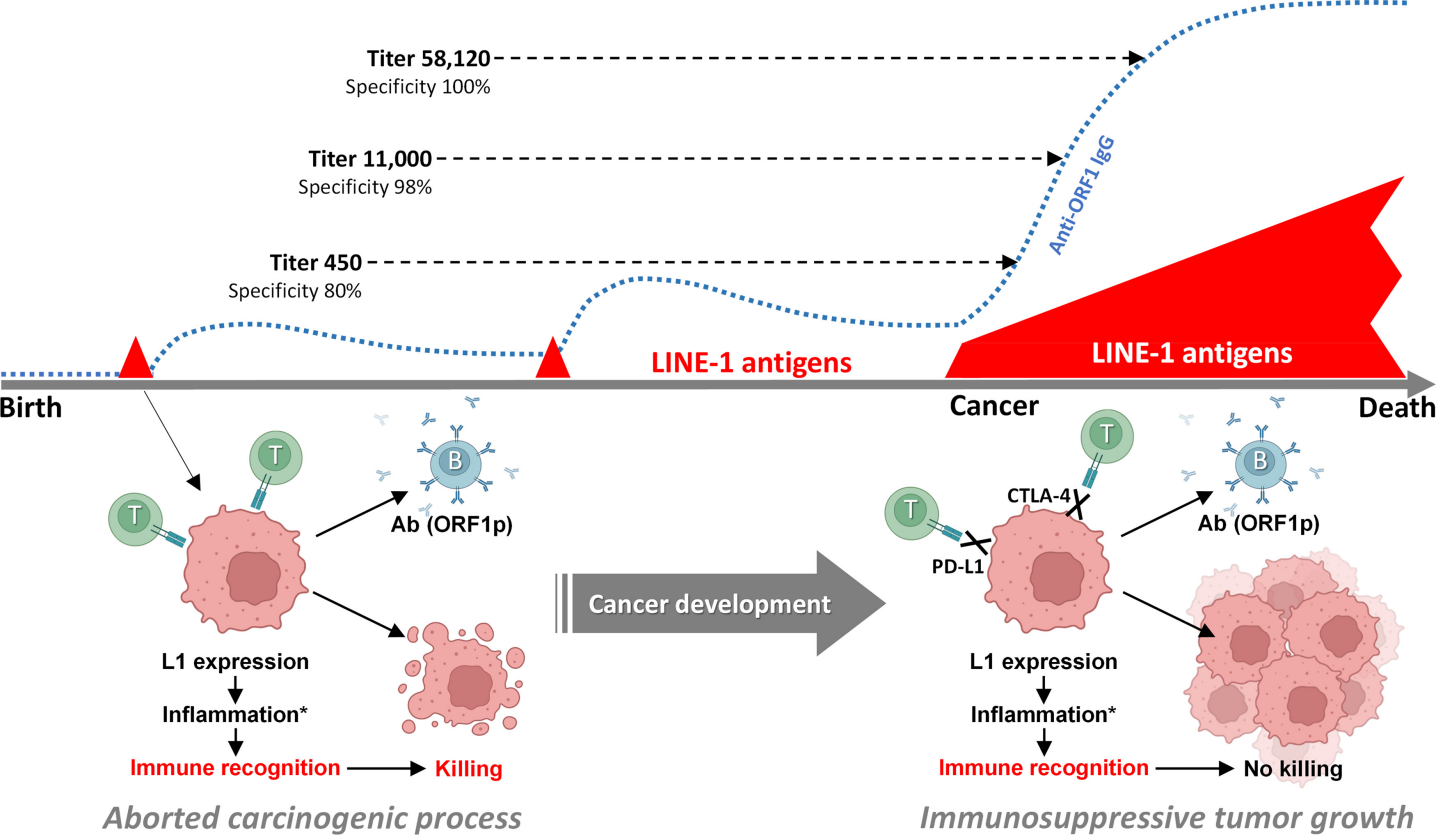
Schematic description of a hypothetical relationship between circulating IgG titers to LINE-1 antigens and cancer susceptibility to immunotherapy by immune checkpoint inhibitors. Presence of low titers of anti-LINE-1 IgG in healthy subjects can be explained by immune response that occurred to precancerous lesions associated with derepression of LINE-1 that were aborted due to effective T-cell response. These events generate memory B cells and the presence of low titers of anti-LINE-1 antibodies even in a cancer-free organism. Development of cancer that passivate T-cell response due to the engagement of immune checkpoint factors PD-L1 and CTLA-4 becomes a growing source of LINE-1 antigens. If this cancer expresses LINE-1 and retains the ability to be recognized by the immune system (e.g., the ability to present antigens), it will then induce elevation of anti-LINE1 IgG levels, which will signal about principal immunoreactivity of the tumor. *This response is stimulated by the cGAS-STING–mediated induction of inflammation.

However, this apparent advantage of anti-L1 antibodies is confounded by their infrequent appearance in healthy volunteers without known cancers or autoimmune diseases. A possible explanation for the presence of detectable levels of anti-ORF1p antibodies in healthy subjects, with a trend of elevation with age, is the presence of an occult disease, or alternatively, that these antibodies remain as a memory of the adaptive immune response to past events associated with the derepression of L1 (e.g., initiated carcinogenic processes intercepted by anticancer defense mechanisms). In fact, it has been demonstrated that L1 derepression can precede cancer development (51). According to this model, the presence of anti-L1 antibodies in healthy individuals may be “remnants” of a past success of antitumor immunity, whereas the strong accumulation of anti-L1 antibody titers in the blood likely signals persistent activation of associated memory B cells by the presence of L1-expressing cells and may reflect an inability of the immune system to effectively eradicate these cells (Fig. 4). These factors imply that elevated anti-L1 antibodies in healthy individuals could potentially signal varying risks of cancer development. They also provide a rationale for future longitudinal studies, which could monitor the dynamics of anti-L1 antibody levels throughout human lifespan, correlated with the timing and type of cancer occurrence.

Only a portion of patients with cancer have elevated levels of anti-L1 IgG antibodies, which precludes the use of anti-ORF1p antibody immunoassay as a negative screening test for cancer. Another apparent limitation of this immunoassay is its inability to distinguish multiple cancer types. In fact, similar frequencies of elevated IgG levels against ORF1p were observed in patients with the five cancer types and this number will likely increase after other cancer types are analyzed. These constrains do not allow us to view anti-ORF1p immunoassay as a stand-alone screening test for cancer presence but rather as a supplementing assay to be included as a component in already used or emerging cancer diagnostic panels in populations at high risk, such as CA125 in patients with ovarian cancer (55) or different versions of liquid biopsy–based assays (56).

Because p53 plays a role in epigenetic repression of L1 (39) and a recent study demonstrated that L1 expression in cancer correlates with p53 mutations (40), it is reasonable to expect a correlation between p53 inactivation in the tumor and the development of an anti-L1 antibody response. We could not directly address this possibility in our study because we did not have information on the p53 status of tumors in patients whose serum samples were analyzed. However, we could test autoantibodies against p53 in our panel, knowing that the development of anti-p53 antibodies usually follows p53 mutations that create highly stable, aberrantly accumulated, and therefore immunogenic proteins. The level of anti-p53 IgG was higher among cancer serum samples with elevated anti-ORF1p IgG (Fig. 1E). The immunogenicity of L1 autoantigens suggests that their effective recognition could be part of evolutionarily developed protective mechanisms against L1 activation.

These considerations suggest another potential clinical application for anti-ORF1p antibody assay: the ability to differentiate between tumors susceptible and resistant to immunotherapy with the inhibitors of immunologic checkpoint factors PD-1 or CTLA-4 that are currently approved for the treatment of multiple cancers (Fig. 4; refs. 57, 58). The lack of reliable predictive tests to define subsets of patients capable of responding to immunotherapy is a significant clinical problem (59, 60). In the context of our hypothesis, high levels of anti-L1 IgG indicate that the immune system “senses” the tumor but cannot effectively eradicate it, suggesting the potential for a high probability of successful immunotherapy with immune checkpoint inhibitors. The potential impact of derepressed retrotransposons on sensitizing tumors to immune checkpoint inhibitor immunotherapy has been proposed (61, 62) and may reflect, in part, the sensitizing effects of DNA hypomethylating agents that are known to stimulate retrotransposon expression (63). Alternatively, low levels of anti-L1 antibodies in patients with advanced L1-positive cancer may be evidence of low tumor immunogenicity and may be predictive of poor immunotherapy performance. While T-lymphocyte responses play a major role in tumor eradication, antibodies to neoantigens are usually harmless to the tumor and, therefore, may serve as long-lasting biomarkers of immune recognition that cannot be translated into an antitumor effect. This hypothesis implies an association between B- and T-cell responses to tumors. The utility of anti-ORF1p immunoassay for this indication could be tested by evaluating the association of anti-L1 antibodies with the response to immunotherapy in NSCL and SCL cancers, which is a subject of our future study.

The obtained results uncover a novel phenomenon—the existence of an autoimmune response to the antigens of L1 family of retrotransposons that can reach exceptionally high levels in part of patients with the five cancer types. Mechanisms underlying broad variability in the scales of responses as well as their physiologic significance remain to be determined.

## Supplementary Material

Supplementary Methods Supplementary Methods include synthesis and purification of recombinant L1 antigens, immunoassays for L1 antigens and peptides derived from ORF1p and ORF2p,and Western immunoblotting protocols

Figure S1 Supplementary Figure S1 shows characteristics of HeLa cells with tetracycline-inducible L1 retrotransposition reporter.

Figure S2 Supplementary Figure S2 shows anti‐ORF1p, anti‐ORF2p IgG titers and anti‐p53 IgG signals in serum samples of patients with 14 cancer types and healthy individuals.

Fig S3 Supplementary Figure S3 shows immunofluorescent staining and Western immunoblotting of lysates of HeLa cells with inducible L1 expression.

Fig S4 Supplementary Figure S4 demonstrates detection of human ORF1p antigen by sandwich ELISA in the presence of human anti‐ORF1p-reactive antibodies.

Fig S5 Supplementary Figure S5 shows anti‐ORF2p IgG titers in the blood of esophageal, lung, pancreatic, ovarian and liver cancer patients.

Fig S6 Supplementary Figure S6 shows correlation of anti‐ORF1p and anti‐ORF2p IgG titers in patients with five cancer types and healthy individuals.

Fig S7 Supplementary Figure S7 shows the ROC curves indicating specificity and sensitivity of anti‐ORF1p immunoassay for 5 cancer types.

Fig S8 Supplementary Figure S8 shows comparison of anti‐ORF1p IgG titers cancer patients with different tobacco smoking history.

Fig S9 Supplementary Figure S9 shows comparison of anti-ORF1p IgG titers in cancer patients with or without exposure to anti‐cancer therapies.

Fig S10 Supplementary Figure S10 shows results of longitudinal study of anti‐ORF1p IgG titers in serum samples from healthy subjects.

Fig S11 Supplementary Figure S11 shows comparison of anti‐ORF1p, anti‐ORF2p and anti‐p53 IgG in blood samples from patients with SLE and healthy individuals.

Table S1 Supplementary Table S1 contains the list of synthetic peptides derived from ORF1p.

Table S2 Supplementary Table S2 shows anti-ORF1p IgG titers and detection of ORF1p by Western blots in a set of selected blood samples of cancer patients.

Table S3 Supplementary Table S3 shows IgG titers against ORF1p-derived peptides in healthy persons, lung, and ovarian cancer patients.

Table S4 Supplementary Table S4 shows the number of samples for five cancer types and distribution by disease stages.

Table S5 Supplementary Table S5 shows the distribution of age between cancer and healthy subjects.

Table S6 Supplementary Table S6 shows results of logistic regression analysis of anti-ORF1 IgG titers in cancer patients relative to healthy individuals after adjusting for age.

Table S7 Supplementary Table S7 shows the results of linear regression analysis of association between ORF1p IgG titers and individual cancer types relative to healthy subjects after adjustment for age.

Table S8 Supplementary Table S8 shows the results of linear regression analysis: association between ORF1p IgG titers and individual cancer types (stages 1-2) relative to healthy subjects after adjustment for age.

Table S9 Supplementary Table S9 shows comparison of anti-ORF1p IgG titers among healthy subjects of different races.

Table S10 Supplementary Table S10 shows gender-specificity of anti-ORF1p IgG titers among healthy subjects and cancer patients.

Table S11 Supplementary Table S11 shows comparison of anti-ORF1p IgG titers between indicated groups of subjects.
