# Supplementary material for: Cancer Relevance of Circulating Antibodies Against LINE-1 Antigens in Humans
Source: Cancer Res Commun. 2023 Nov 8;3(11):2256–67. doi: 10.1158/2767-9764.CRC-23-0289 (PMC10631453; doi:10.1158/2767-9764.CRC-23-0289)
Supplement: Supplementary Methods — include synthesis and purification of recombinant L1 antigens, immunoassays for L1 antigens and peptides derived from ORF1p and ORF2p,and Western immunoblotting protocols [file crc-23-0289-s01.pdf]

Recombinant L1 antigens synthesis and purification

The protein-coding DNA sequence corresponding to human L1 ORF1p described in Uniport Entry Q9UN81 (LORF1_HUMAN) was custom-synthesized with codon optimization for expression in E. coli (GenScript Biotech) and cloned into the modified pET-49b(+) expression vector (Novagen) with a C-terminal His6-tag under control of the T7 promoter. Overexpression in *E. coli* strain T7 Express Iq (NEB, Cat. # C2833H) was optimized to yield ~100 mg/L of recombinant ORF1p-His6 (346 aa; 41.1 kDa) in the form of inclusion bodies (IB). Cells harvested by centrifugation were resuspended in lysis buffer (20 mM HEPES pH 7.0; 0.5M NaCl; 0.5% Brij-35) and lysed by sonication. Insoluble fraction collected after centrifugation of the crude lysate was extracted with 7M Urea-based extraction/ capturing buffer (50mM Tris-HCl, pH 8.5; 250mM NaCl; 7M Urea; 20mM Imidazole; 0.15% Brij35; 2.5mM β-mercaptoethanol; 1/20 volume of expression culture). Capturing of ORF1p-His6 protein was performed using Immobilized-Metal Affinity Chromatography (IMAC) by loading of 7M Urea Extract on 5 mL HisTrap FF FPLC column (GE Healthcare) at 0.5 mL/minute flow rate followed by washing with 10 column volumes (CV) of the same 7M Urea-based buffer. Partial in-column refolding by washing with 10 CV of 4M Urea-based buffer with 0.5M NaCl and 0.3% Brij35 was followed by gradient elution with 20-300mM imidazole in 4M Urea-based buffer. Pooling of the main peak fractions yielded highly purified ORF1p protein (~90% by SDS-PAGE analysis) without appreciable host cell proteins (HCPs) impurities. Two-step dialysis assisted transferring of recombinant ORF1p protein to storage buffer. Initial dialysis against storage buffer containing 2M Urea was followed by dialysis against storage buffer: 50mM Tris-HCl, pH 8.0; 0.5M NaCl; 2mM DTT; 0.05% Tween80; 5% Glycerol'. Sterile-filtered final protein sample with concentration adjusted to 1mg/ml was flash-frozen in aliquots in low protein binding (LPB) plastic vials before storage at -80°C.

The protein-coding DNA sequence corresponding to fragment 367-771 of human L1 ORF2p described in Uniport Entry O00370 (LORF2_HUMAN); or as human NAG13 protein (GenBank ID: AAG27485.1) was custom-synthesized with codon optimization for expression in E. coli (GenScript Biotech). Cloning and expression of 367-771 fragment (413 aa; 47.6 kDa) of human L1 ORF2p was performed according to the same protocol as described above for human ORF1p, with exception of using the Size-Exclusion Chromatography (SEC) as final purification step after IMAC purification/refolding. HiLoad 16/600 Superdex-200 FPLC column (GE Healthcare) was equilibrated with 50mM Tris-HCl, pH 8.5; 2M Urea; 0.5M NaCl; 0.15% Brij35; 2.5mM DTT. SEC-eluted samples were subject to buffer exchange on Fast-Desalt FPLC column HiPrep 26/10 (GE Healthcare) in 50mM Tris-HCl, pH 8.0; 0.3M NaCl; 0.5mM DTT; 0.05% Tween80; 5% Glycerol, flash frozen and stored at -80°C.

Anti-ORF1p and anti-ORF2p immunoassays

The anti-ORF1p or anti-ORF2p assays were designed to detect anti-ORF1p or anti-ORF2p antibodies using an indirect ELISA method performed in plates coated with ORF1p or a fragment of ORF2p proteins encoded by an L1 retroelement capable of expression in humans. The titers of anti-ORF1p reactive IgG antibodies in human serum were measured in assay plates coated with a full-length ORF1p protein as antigen; the titers of anti-ORF2p reactive IgG antibodies in human serum were measured in assay plates coated with a RT-domain (amino acids residues 367 -771) as antigen as described below.

Nunc MaxiSorp clear 96-well plates were coated with the antigens at 2 µg/mL in phosphate-buffered saline (PBS), 50 µL per well overnight at 4°C. The plates without adsorbed antigen, no-antigen control plates, served to evaluate non-specific antibody binding to plastics. The assay plates were washed with PBS, containing 0.05% Tween 20 (PBST) then blocked with Blocker Casein in PBS, supplemented with 0.05% Tween 20 (Assay Buffer). Human serum samples were serially diluted in Assay Buffer, starting with a 100-fold minimum required dilution to a 218,700-fold final dilution and were added

to the ORF1p, ORF2p and no-antigen control assay plates in duplicates, 50 µL per well. After incubation at room temperature for 2 hours, the assay plates were washed with PBST, and the bound ORF1p- and ORF2p-reactive IgG antibodies were detected with anti-human IgG-HRP (Jackson ImmunoResearch) secondary antibody conjugates diluted 2,000-fold in Assay Buffer for 1 hour at room temperature. The plates were washed six times with PBST and incubated with 50 µL of 1-Step Ultra TMB-ELISA Substrate Solution. Peroxidase reactions were stopped with 25 µL 1% hydrochloric acid after 30-minute incubation at room temperature and optical densities at 450 nm (OD450) were measured using a plate reader (Tecan Infinite M1000 Pro). Antibody titers defined as the highest sample dilution that produce positive OD450 reading above a cutpoint value (2-fold OD450 in the wells without test sample) were calculated by a log-linear approximation.

Anti-p53 and anti-ORF1p peptide antibody ELISA

Recombinant Human Tumor Protein p53 (RayBiotech, Code 230-00639-10) was diluted to 1 µg/mL in a 2M urea/PBS coating buffer; or each of seventeen ORF1p 25-mer peptides conjugated with BSA were diluted to 2 µg/mL in PBS coating buffer and added to Black MaxiSorp 96-well assay plates (50 µL/well). The coating buffer without p53 or peptides was added to no-antigen control plates. After overnight incubation at 4°C, assay plates were washed with PBST and blocked with Blocker Casein in PBS, 0.05% Tween 20 (Assay Buffer). Human serum samples were diluted 300-fold in Assay Buffer and added to the p53 or peptides and no-antigen control assay plates, 50 µL/well. After incubation at room temperature for 2 hours, the assay plates were washed with PBST, and the bound p53-reactive IgG antibodies were detected with anti-human IgG-HRP (Jackson ImmunoResearch) diluted 1,000-fold in Assay Buffer for 1 hour at room temperature. The plates were washed with PBST and incubated with 50 µL QuantaBlu fluorogenic peroxidase substrate (ThermoFisher) for 15 minutes at room temperature. Fluorescence (RFU) was measured at an excitation wavelength of 320 nm and an emission wavelength of 420 nm using a plate reader (Tecan Infinite M1000 Pro). Fluorescence measured in no-antigen control wells was subtracted from the corresponding RFU values obtained from p53- or peptides-coated wells.

Western immunoblotting

Plates with HeLa tet-L1/GLucAI cells +/-Dox were placed on ice and the cells were washed with ice-cold PBS. After PBS aspiration, cells were sonicated in ice cold RIPA buffer (Sigma) plus protease inhibitors (PIC) (Sigma) and cleared by centrifugation in a Beckman tabletop (20-minute, 12,000 rpm, 4°C). The protein concentrations of lysates were determined with the Quick start Bradford reagent (Bio-Rad), using bovine serum albumin (BSA) (Sigma) as a standard. Cells lysates were separated by BioRad mini protean TGX gels 4-20%, 15 µg of total cell protein were loaded per well. Precision plus dual color protein molecular weight markers (Bio-Rad) were also loaded. For Western blots, proteins were then transferred to Immun-Blot PVDF membranes (Bio-Rad). After blocking in 2.5% non-fat dry milk (RPI) in TBST (10 mM Tris-HCl (ThermoFisher) pH 7.5, 150 mM NaCl (ThermoFisher), 0.1% Tween-20 (Sigma)) for 1 hour at room temperature, blots were incubated with primary antibodies against ORF1p overnight at 4°C with constant shaking: 1) anti-ORF1p IgG high-titers human serum samples in 1% non-fat dry milk (RPI) TBST in dilution 1:1,000-1000,000, followed by horseradish peroxidase (HRP)-conjugated secondary anti-human IgG antibodies (1:10,000 dilution, Sera Care) 1 hour at room temperature or 2) rabbit anti-human ORF1p polyclonal serum in 1% non-fat dry milk (RPI) in TBST in dilution 1:1,000, followed by horseradish peroxidase (HRP)-conjugated secondary anti-mouse IgG antibodies (1:4,000 dilution, Santa Cruz) 1 hour at room temperature. Membranes were washed three times with TBST, then developed by ECL (Perkin-Elmer) and documented using the ChemiDoc MP Imaging System (BioRad).
