# Supplementary material for: Cancer Relevance of Circulating Antibodies Against LINE-1 Antigens in Humans
Source: Cancer Res Commun. 2023 Nov 8;3(11):2256–67. doi: 10.1158/2767-9764.CRC-23-0289 (PMC10631453; doi:10.1158/2767-9764.CRC-23-0289)
Supplement: Figure S1 — Supplementary Figure S1 shows characteristics of HeLa cells with tetracycline-inducible L1 retrotransposition reporter. [file crc-23-0289-s02.pdf]

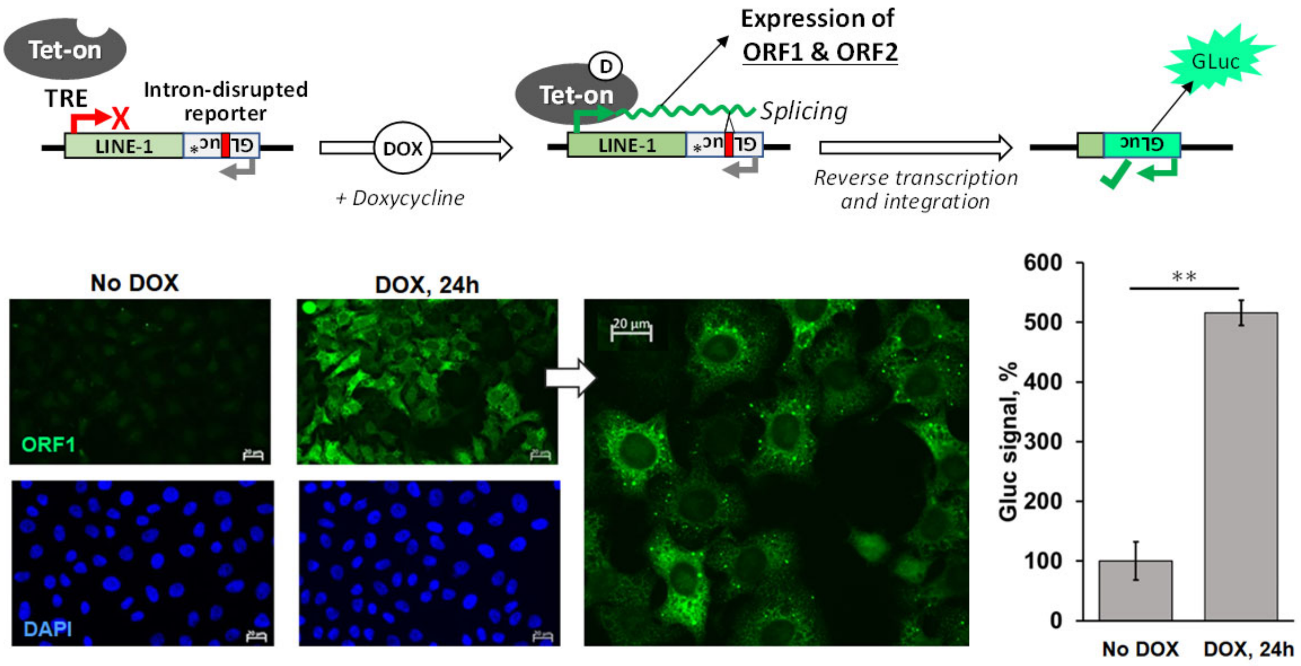

Figure S1. Characteristics of HeLa tet-L1/GLucAI retrotransposition reporter cells. A schematic of the doxycycline-inducible L1 cassette stably integrated into HeLa cells (top panel). Fluorescent microphotographs depict the induction of human ORF1p in HeLa tet-L1/GLucAI cells following a 48-hour induction with 100 ng/mL doxycycline, as demonstrated by punctate cytoplasmic immunoreactivity by rabbit anti-human ORF1p antibodies (green) with DAPI nuclear counterstain (blue) (bottom left). Following a 48-hour exposure to doxycycline (400 ng/mL), an induction of secreted *Gaussia* luciferase activity (a readout for L1-mediated retrotransposition) was observed (bottom right). Mean and standard deviation of normalized data (compared to non-induced control) are depicted. Statistics were calculated by Student's t-test (unpaired, two-tailed); ** p-value < 0.01.
