# Supplementary material for: Cancer Relevance of Circulating Antibodies Against LINE-1 Antigens in Humans
Source: Cancer Res Commun. 2023 Nov 8;3(11):2256–67. doi: 10.1158/2767-9764.CRC-23-0289 (PMC10631453; doi:10.1158/2767-9764.CRC-23-0289)
Supplement: Figure S2 — Supplementary Figure S2 shows anti‐ORF1p, anti‐ORF2p IgG titers and anti‐p53 IgG signals in serum samples of patients with 14 cancer types and healthy individuals. [file crc-23-0289-s03.pdf]

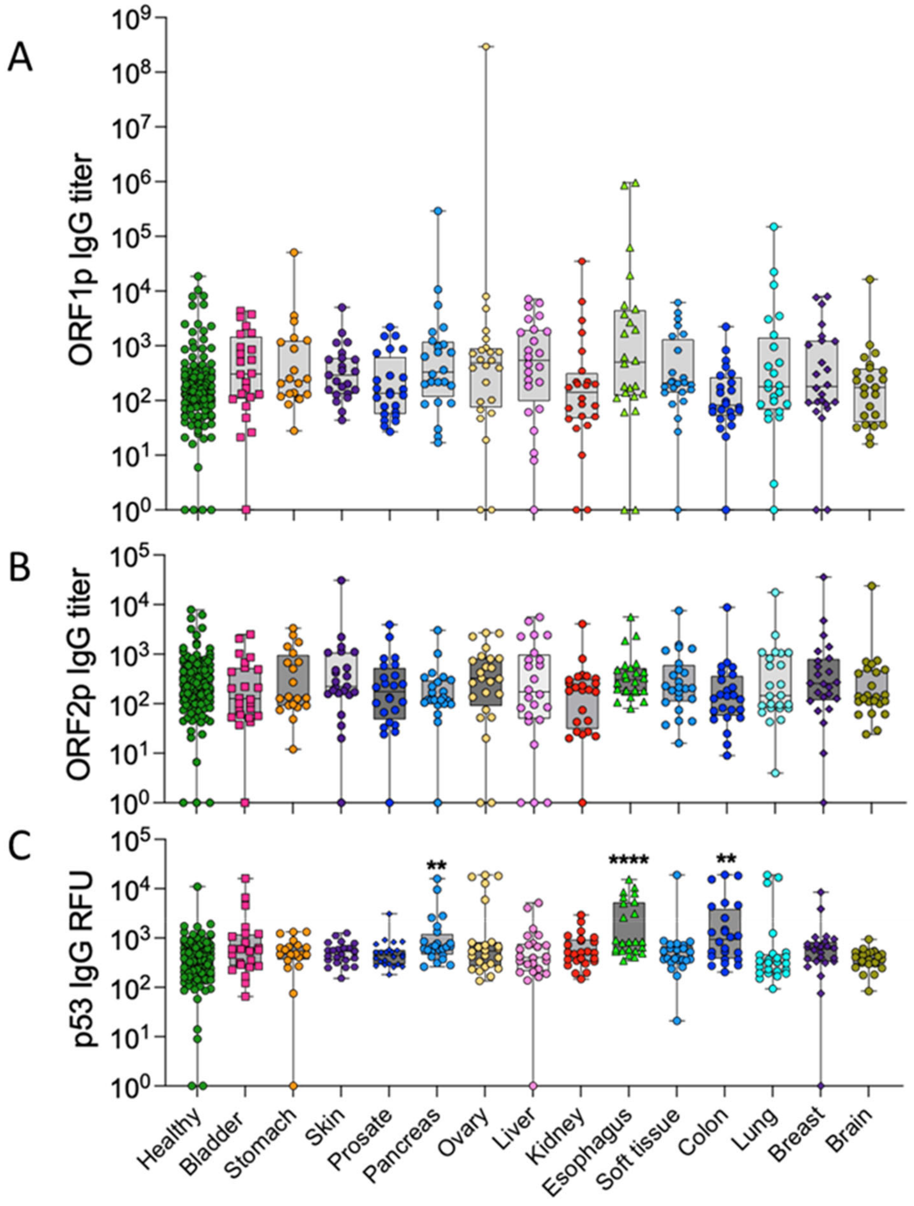

Figure S2. Anti-ORF1p, anti-ORF2p IgG titers and anti-p53 IgG signals in serum samples of patients with 14 cancer types and healthy individuals. A. Boxplots for anti-ORF1p IgG titers, **B.** anti-ORF2p IgG titers and **C.** anti-p53 IgG signals represented by median with range and individual values. Statistics were calculated by Dunn's multiple comparison test with adjusted p-value for anti-ORF1p, anti-ORF2p IgG titers and anti-p53 IgG signals in ELISA for serum samples representing 14 solid cancer types: ovarian (N=24), breast (N=24), lung cancer (N=24), colorectal (N=24), esophageal (N=24), renal (N=24), liver (N=24), pancreatic (N=24), prostatic (N=24), gastric (N=20), bladder (N=24) cancer, soft tissue sarcoma (N=24), melanoma (N=23), glioblastoma (N=24), vs. healthy individuals (N=137). **, $P < 0.01$; ****, $P < 0.0001$
