# Supplementary material for: Cancer Relevance of Circulating Antibodies Against LINE-1 Antigens in Humans
Source: Cancer Res Commun. 2023 Nov 8;3(11):2256–67. doi: 10.1158/2767-9764.CRC-23-0289 (PMC10631453; doi:10.1158/2767-9764.CRC-23-0289)
Supplement: Fig S3 — Supplementary Figure S3 shows immunofluorescent staining and Western immunoblotting of lysates of HeLa cells with inducible L1 expression. [file crc-23-0289-s04.pdf]

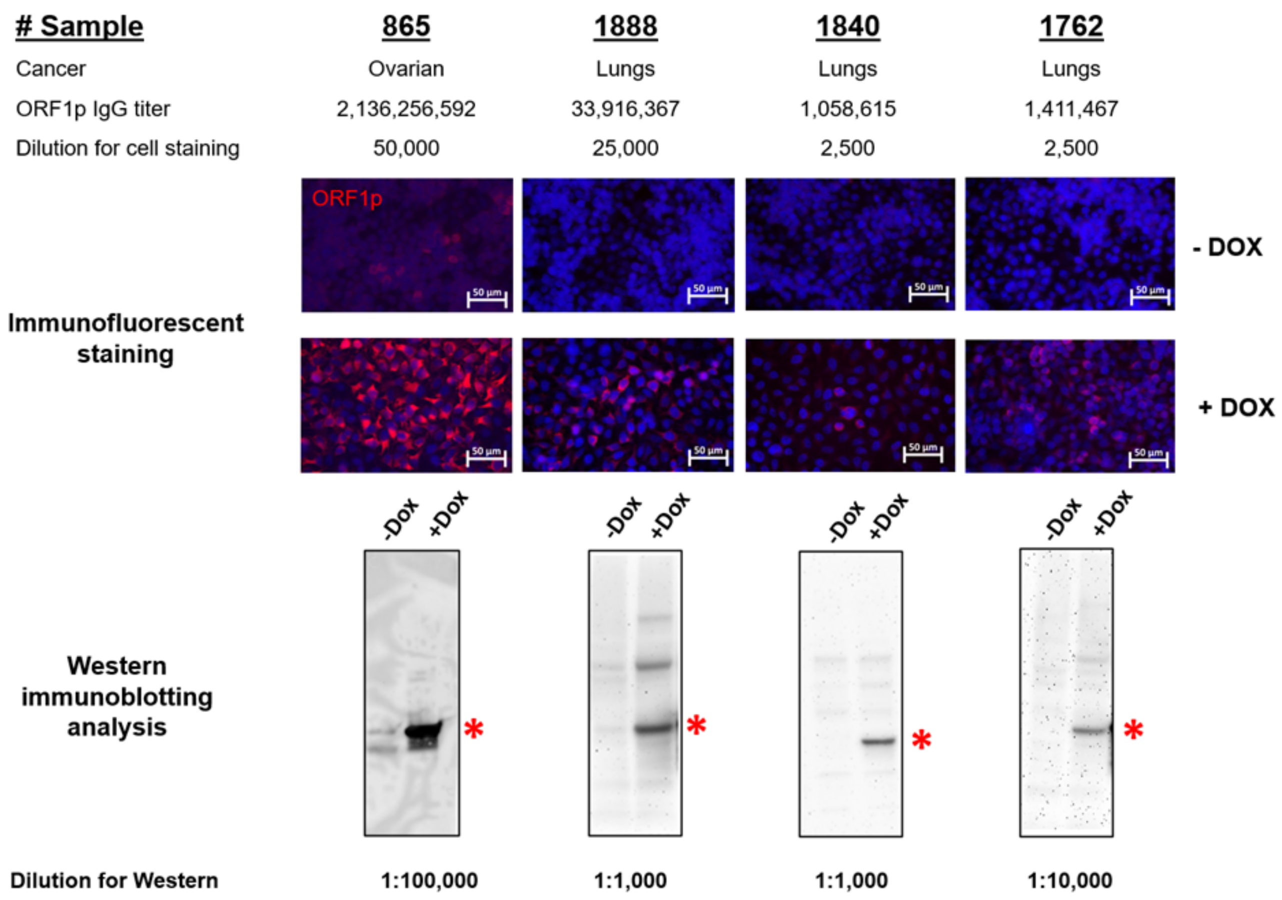

Figure S3. Autoimmune antibodies to L1 ORF1p in serum samples from cancer patients detected by two immunoassays. The results of immunofluorescent staining (upper panel) and immunoblot analysis (lower panel) of HeLa cells transduced with Tet-inducible L1/GlucAI reporter construct with (+ Dox) and without (-Dox) L1 induction by doxycycline for 24 hours. The panels represent staining with diluted serum samples from ovarian and lung cancer patients scored positive in anti-ORF1p immunoassay (#865, #1888, #1840, #1762). The asterisk on the lower panel corresponds to 40-kDa band of ORF1p recombinant protein standard.
