# Supplementary material for: Cancer Relevance of Circulating Antibodies Against LINE-1 Antigens in Humans
Source: Cancer Res Commun. 2023 Nov 8;3(11):2256–67. doi: 10.1158/2767-9764.CRC-23-0289 (PMC10631453; doi:10.1158/2767-9764.CRC-23-0289)
Supplement: Fig S4 — Supplementary Figure S4 demonstrates detection of human ORF1p antigen by sandwich ELISA in the presence of human anti‐ORF1p-reactive antibodies. [file crc-23-0289-s05.pdf]

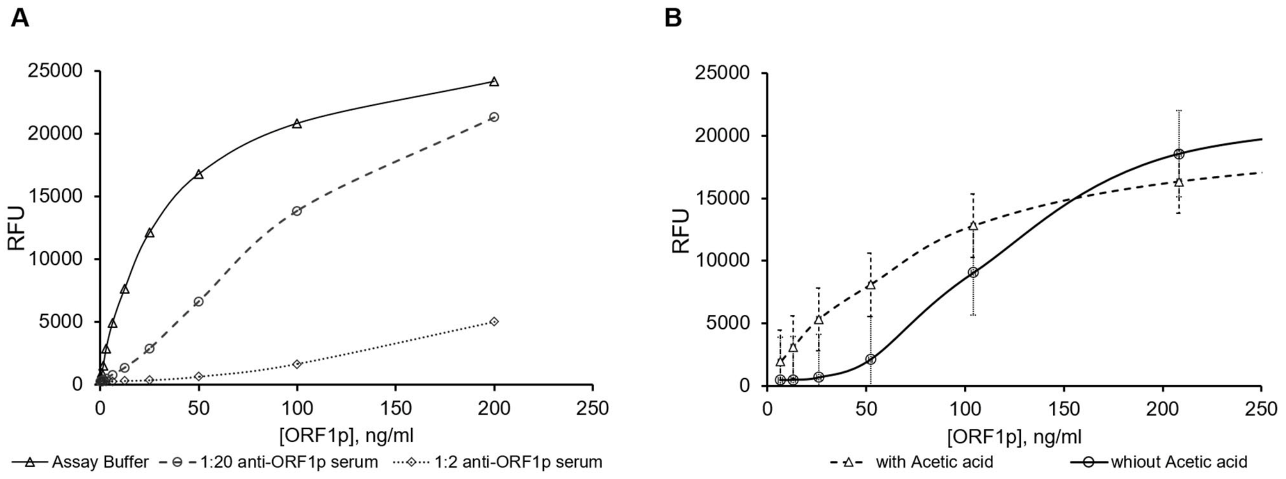

Figure S4. Detection of human ORF1p antigen by sandwich ELISA in the presence of human anti-ORF1p-reactive antibodies. A. Concentration dependence of anti-ORF1p-reactive antibodies from human serum on the detection of human ORF1p antigen standard curve by sandwich ELISA. **B.** Human ORF1p antigen calibration plot in serum with added human anti-ORF1p reactive antibodies with and without acid dissociation detected by sandwich ELISA.
