# Supplementary material for: Cancer Relevance of Circulating Antibodies Against LINE-1 Antigens in Humans
Source: Cancer Res Commun. 2023 Nov 8;3(11):2256–67. doi: 10.1158/2767-9764.CRC-23-0289 (PMC10631453; doi:10.1158/2767-9764.CRC-23-0289)
Supplement: Fig S5 — Supplementary Figure S5 shows anti‐ORF2p IgG titers in the blood of esophageal, lung, pancreatic, ovarian and liver cancer patients. [file crc-23-0289-s06.pdf]

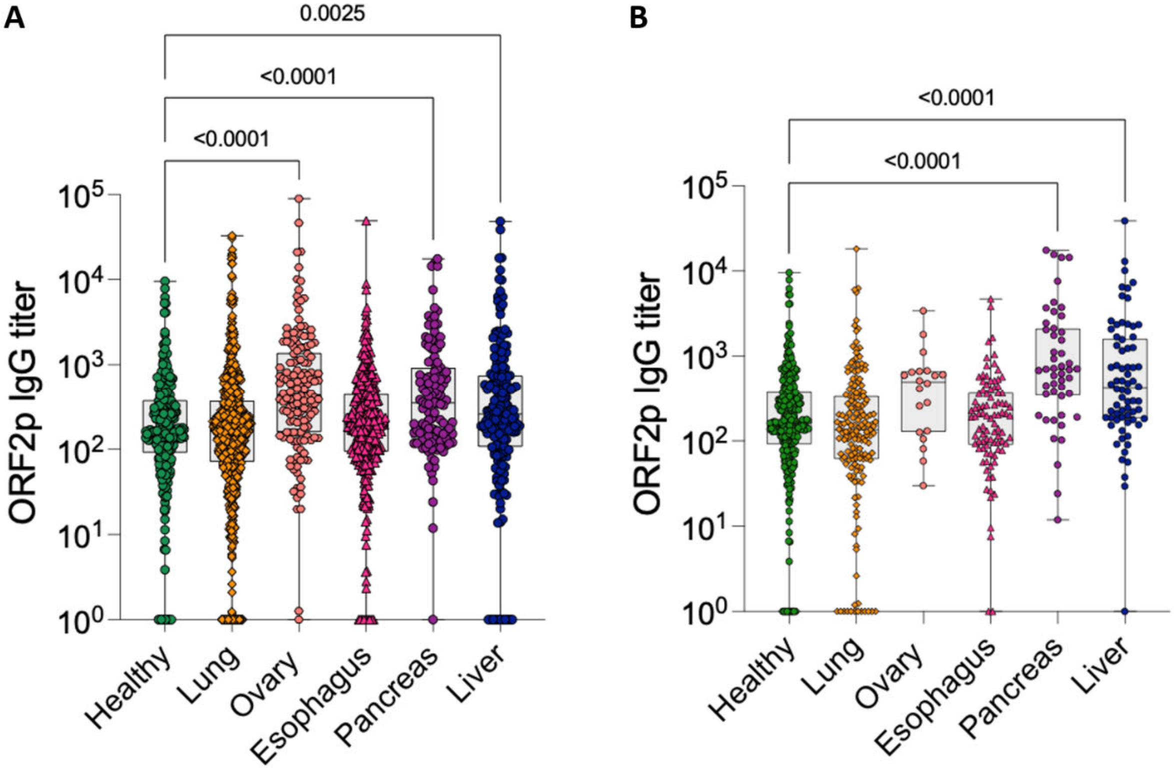

Figure S5. Levels of anti-ORF2p IgG in serum samples of patients with esophageal, lung, pancreatic, ovarian and liver cancer. A. Detection of anti-ORF2p IgG titers in serum samples of patients with indicated cancer types (all stages). Sample size: healthy (N=274), lung (N=707), ovary (N=150), esophagus (N=377), pancreas (N=124), and liver (N=217) **B.** The same as panel A, except only samples from patients with cancer stages 1 and 2 are shown. Sample size: healthy (N=274), lung (N=169), ovary (N=20), esophagus (N=92), pancreas (N=48), and liver (N=70). Boxplots for ORF2p IgG titers for five selected cancer types depicting median with range and individual values. Statistics were calculated by Dunn's multiple comparison test with multiplicity-adjusted p-values for anti-ORF2p IgG titers in ELISA for five cancer categories vs. healthy individuals. All p-values < 0.05 are indicated.
