# Supplementary material for: Cancer Relevance of Circulating Antibodies Against LINE-1 Antigens in Humans
Source: Cancer Res Commun. 2023 Nov 8;3(11):2256–67. doi: 10.1158/2767-9764.CRC-23-0289 (PMC10631453; doi:10.1158/2767-9764.CRC-23-0289)
Supplement: Fig S6 — Supplementary Figure S6 shows correlation of anti‐ORF1p and anti‐ORF2p IgG titers in patients with five cancer types and healthy individuals. [file crc-23-0289-s07.pdf]

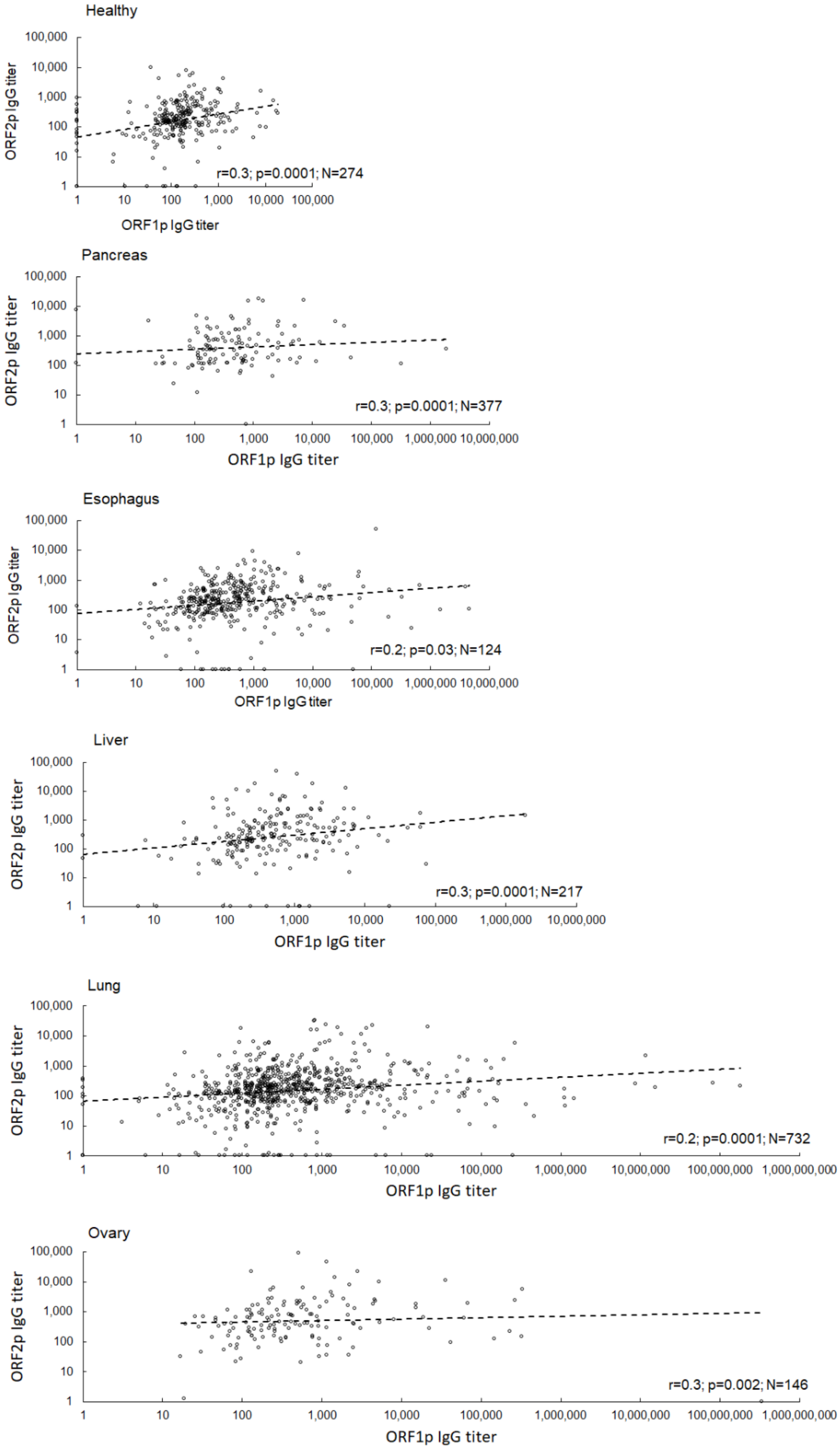

Figure S6. Spearman's correlation analysis of anti-ORF1p and anti-ORF2p IgG titers in five cancer types and healthy individuals.
