# Supplementary material for: Cancer Relevance of Circulating Antibodies Against LINE-1 Antigens in Humans
Source: Cancer Res Commun. 2023 Nov 8;3(11):2256–67. doi: 10.1158/2767-9764.CRC-23-0289 (PMC10631453; doi:10.1158/2767-9764.CRC-23-0289)
Supplement: Fig S7 — Supplementary Figure S7 shows the ROC curves indicating specificity and sensitivity of anti‐ORF1p immunoassay for 5 cancer types. [file crc-23-0289-s08.pdf]

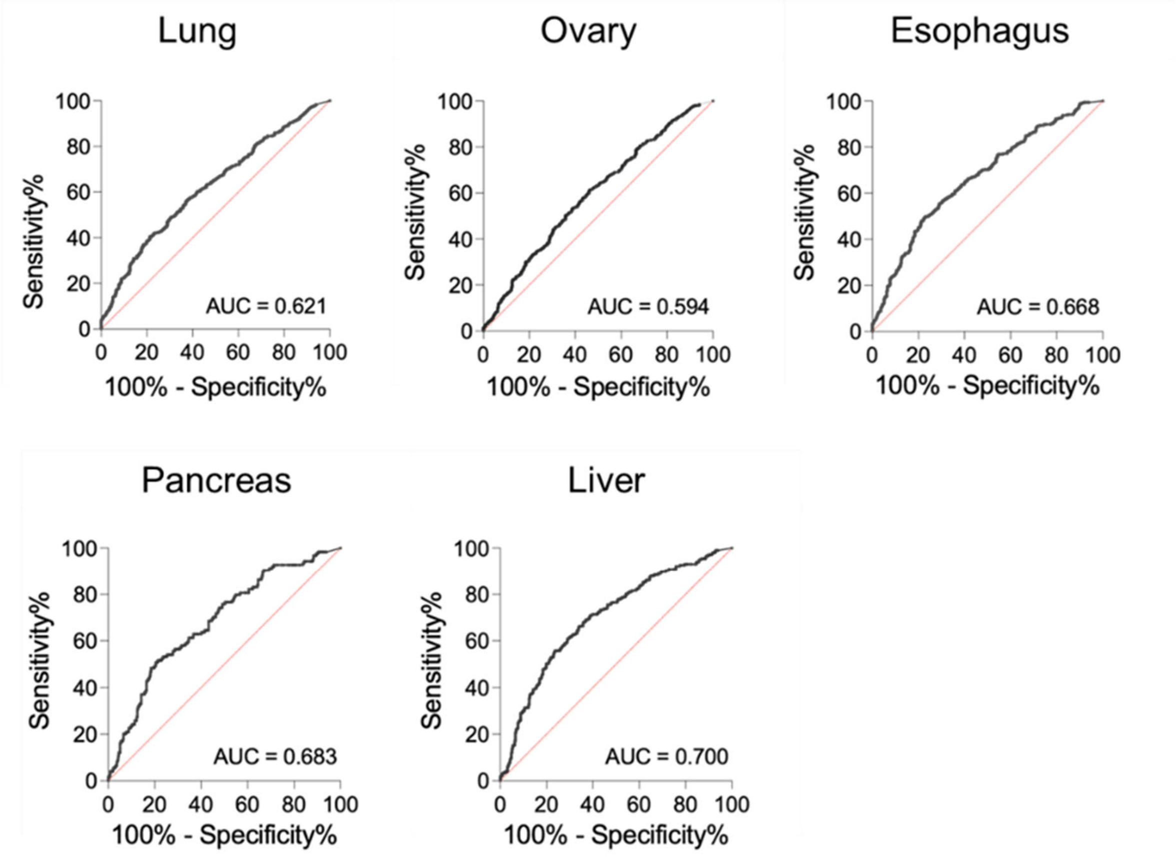

Figure S7. The ROC curves indicating specificity and sensitivity of anti-ORF1p immunoassay for patients with five indicated cancer types vs healthy subjects. Lung (N=908), ovary (N=993), esophagus (N=377), pancreas (N=124), liver (N=217) cancers and healthy subjects (N=352). All p-values < 0.0001.
