# Supplementary material for: Cancer Relevance of Circulating Antibodies Against LINE-1 Antigens in Humans
Source: Cancer Res Commun. 2023 Nov 8;3(11):2256–67. doi: 10.1158/2767-9764.CRC-23-0289 (PMC10631453; doi:10.1158/2767-9764.CRC-23-0289)
Supplement: Fig S8 — Supplementary Figure S8 shows comparison of anti‐ORF1p IgG titers cancer patients with different tobacco smoking history. [file crc-23-0289-s09.pdf]

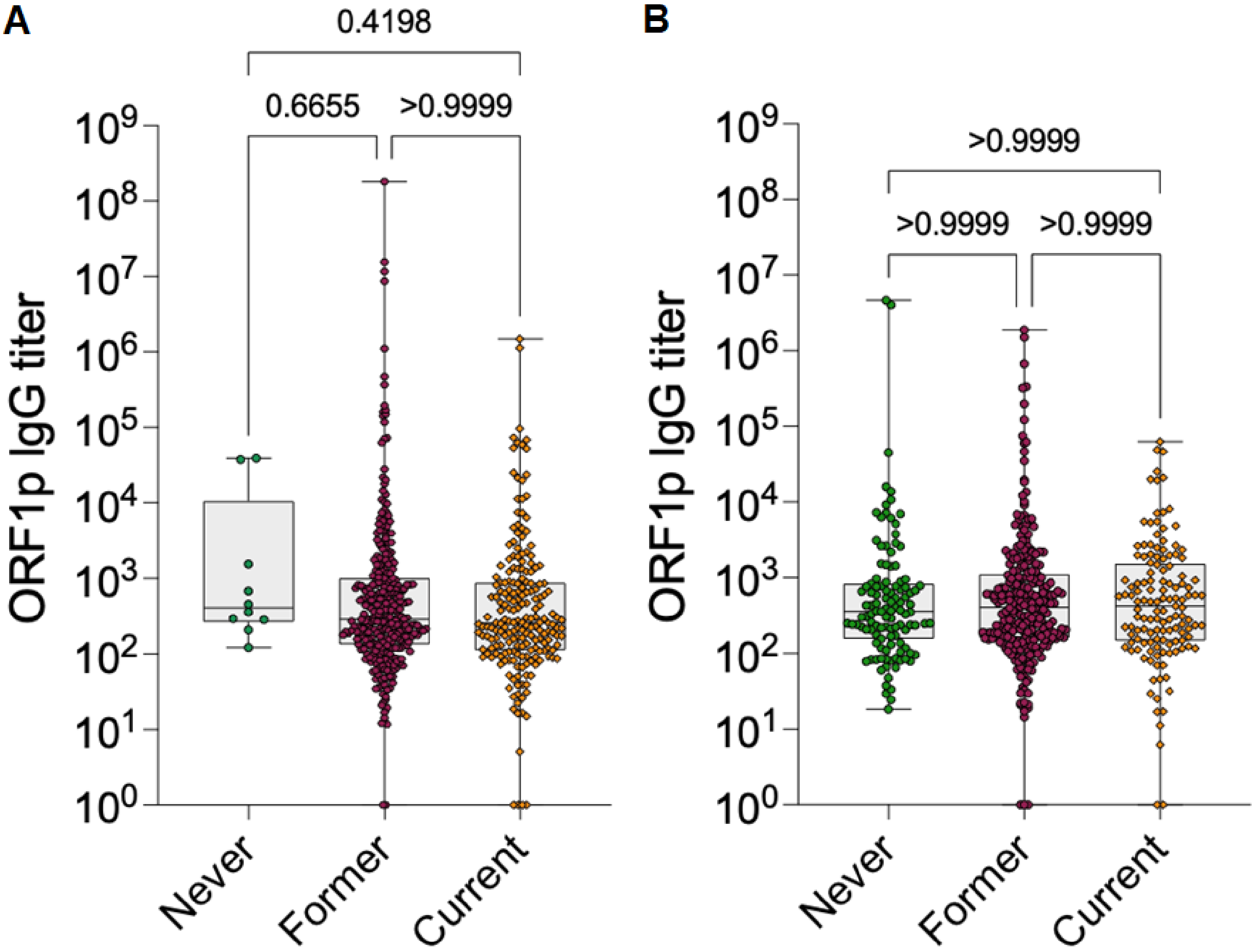

Figure S8. Comparison of anti-ORF1p IgG titers cancer patients with different tobacco smoking history. A. Lung cancer: former smokers (N=321), never smokers (N=10), current smokers (N=205). **B**. Combined cancer group: former smokers (N=266), never smokers (N=115), current smokers (N=133). Boxplots for anti-ORF1p IgG titers for patients with lungs cancer grouped by smoking status, depicting the minimum, first quartile, median, third quartile, maximum and individual titer values. Statistics were calculated by Dunn's multiple comparison test with adjusted p-values for anti-ORF1p IgG titers determined by ELISA for three groups: former smokers, current smokers, and never smokers.
