# Supplementary material for: Cancer Relevance of Circulating Antibodies Against LINE-1 Antigens in Humans
Source: Cancer Res Commun. 2023 Nov 8;3(11):2256–67. doi: 10.1158/2767-9764.CRC-23-0289 (PMC10631453; doi:10.1158/2767-9764.CRC-23-0289)
Supplement: Fig S9 — Supplementary Figure S9 shows comparison of anti-ORF1p IgG titers in cancer patients with or without exposure to anti‐cancer therapies. [file crc-23-0289-s10.pdf]

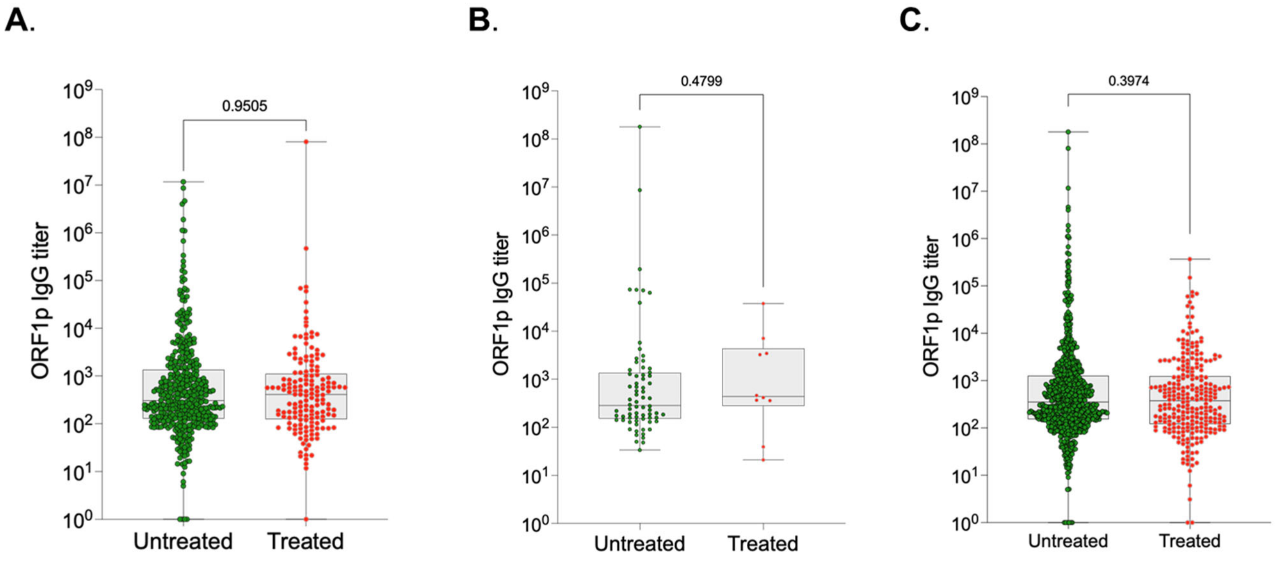

Figure S9. Cross-sectional assessment of anti-ORF1p IgG titers in cancer patients with or without exposure to anti-cancer therapies. Anti-ORF1p IgG titers in cancer patients combined from four cancer types (lungs, pancreatic, esophageal, and liver). **A.** Radiation therapy: untreated (N=442), treated (N=161). **B.** Immunotherapy: untreated (N=72), treated (N=10). **C.** Chemotherapy: untreated (N=771), treated (N=266). Boxplots for anti-ORF1p IgG titers combined for patients with four cancer types grouped by therapy, depicting the minimum, first quartile, median, third quartile, maximum and individual titer values. Statistics were calculated by Mann-Whitney test for anti-ORF1p IgG titers determined by ELISA for treated vs. untreated cancer patients. All p-values > 0.05.
