# Supplementary material for: Cancer Relevance of Circulating Antibodies Against LINE-1 Antigens in Humans
Source: Cancer Res Commun. 2023 Nov 8;3(11):2256–67. doi: 10.1158/2767-9764.CRC-23-0289 (PMC10631453; doi:10.1158/2767-9764.CRC-23-0289)
Supplement: Fig S10 — Supplementary Figure S10 shows results of longitudinal study of anti‐ORF1p IgG titers in serum samples from healthy subjects. [file crc-23-0289-s11.pdf]

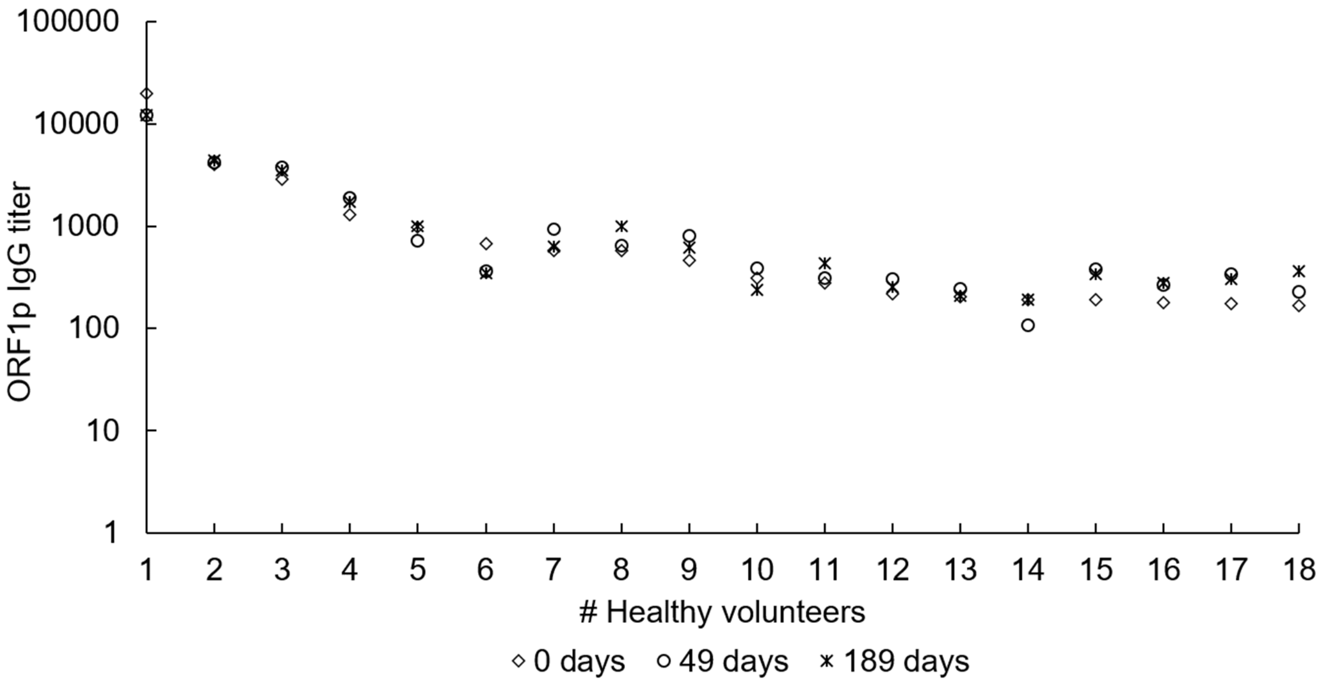

Figure S10. Longitudinal study of anti-ORF1p IgG titers in serum samples from healthy volunteers. Blood was drawn from 18 healthy individuals (66±10 (mean ±SD) years of age) on day 0, 49, and 189. Anti-ORF1 IgG titers were determined by ELISA. The average coefficient of variation for titer measurements from individual volunteers was 25%.
