# Supplementary material for: Cancer Relevance of Circulating Antibodies Against LINE-1 Antigens in Humans
Source: Cancer Res Commun. 2023 Nov 8;3(11):2256–67. doi: 10.1158/2767-9764.CRC-23-0289 (PMC10631453; doi:10.1158/2767-9764.CRC-23-0289)
Supplement: Fig S11 — Supplementary Figure S11 shows comparison of anti‐ORF1p, anti‐ORF2p and anti‐p53 IgG in blood samples from patients with SLE and healthy individuals. [file crc-23-0289-s12.pdf]

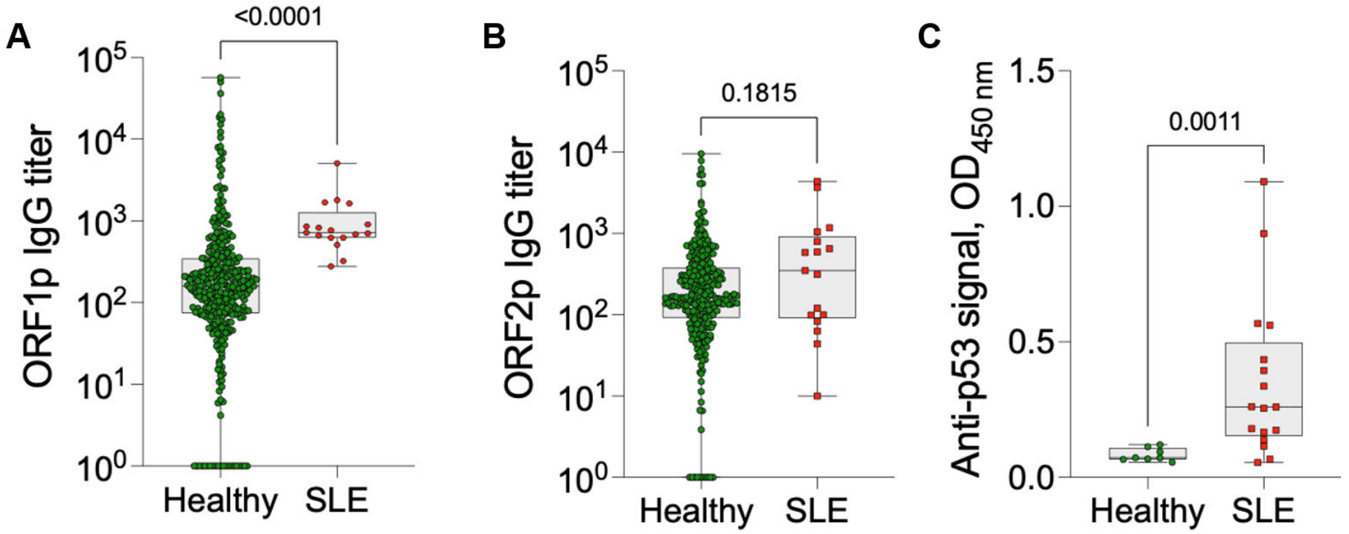

Figure S11. Comparison of anti-ORF1p, anti-ORF2p and anti-p53 IgG levels in blood samples from patients with SLE and healthy individuals. A. Anti-ORF1p IgG titers in samples of SLE patients (N=17) vs. healthy control samples (N=352) determined by ELISA. **B.** Anti-ORF2p IgG titers in samples of SLE patients (n=17) vs. healthy control (N=274) determined by ELISA. **C.** Anti-p53 signals in **"**p53 Autoantibody ELISA Kit (Human)" (Dianova). Statistics were calculated by Mann-Whitney U-test, p-value < 0.05 is considered significant.
