# Supplementary material for: Cancer Relevance of Circulating Antibodies Against LINE-1 Antigens in Humans
Source: Cancer Res Commun. 2023 Nov 8;3(11):2256–67. doi: 10.1158/2767-9764.CRC-23-0289 (PMC10631453; doi:10.1158/2767-9764.CRC-23-0289)
Supplement: Table S1 — Supplementary Table S1 contains the list of synthetic peptides derived from ORF1p. [file crc-23-0289-s13.pdf]

Table S1. The list of peptides covering ORF1p used for epitope mapping of anti-ORF1p IgG. Overlapping amino acid stretches are shown in red; added C-terminal cysteine residues are shown in italics.

#	Peptide (N → C termini)
1	MGKKQNRKTGNSKTQSASPPPKERS*C*
2	PKERSSSPATEQSWMENDFDELREE*C*
3	ELREEGFRRSNYSELREDIQTKGKE*C*
4	TKGKEVENFEKNLEECITRITNTEKC
5	TNTEKCLKELMELKTKARELREECR*C*
6	REECRSLRSRCDQLEERVSAMEDEM*C*
7	MEDEMNEMKREGKFREKRIKRNEQS*C*
8	RNEQSLQEIWDYVKRPNLRLIGVPE*C*
9	IGVPESDVENGTKLENTLQDIIQEN*C*
10	IIQENFPNLARQANVQIQEIQRTPQ*C*
11	QRTPQRYSSRRATPRHIIVRFTKVE*C*
12	FTKVEMKEKMLRAAREKGRVTLKGK*C*
13	TLKGKPIRLTADLSAETLQARREWG*C*
14	RREWGPIFNILKEKNFQPRISYPAK*C*
15	SYPAKLSFISEGEIKYFIDKQMLRD*C*
16	QMLRDFVTTRPALKELLKEALNMER*C*
17	LNMERNNRYQPLQNHAKM*C*
