# Supplementary material for: Cancer Relevance of Circulating Antibodies Against LINE-1 Antigens in Humans
Source: Cancer Res Commun. 2023 Nov 8;3(11):2256–67. doi: 10.1158/2767-9764.CRC-23-0289 (PMC10631453; doi:10.1158/2767-9764.CRC-23-0289)
Supplement: Table S2 — Supplementary Table S2 shows anti-ORF1p IgG titers and detection of ORF1p by Western blots in a set of selected blood samples of cancer patients. [file crc-23-0289-s14.pdf]

Table S2. Anti-ORF1p IgG titers and Western blot (WB)-based detection of ORF1p in selected set of serum sample. Detection of the 40-kDa band on WB of doxycycline-induced HeLa tet-L1/GLucAI cell lysates stained by anti-ORF1p human IgG from the serum samples of cancer patients and healthy volunteers.

# Sample	Cancer type	ORF1p IgG titers	ORF1p on WB
1510	Lung	180,046,944	+
1206		80,557,768	+
1888		33,916,367	+
1390		11,669,656	+
373		5,320,280	+
1132		1,482,912	+
1762		1,411,483	+
1376		1,129,245	+
1050		1,103,532	-
1840		1,058,615	+
1189		1,045,443	+
1025		469,697	+
990		369,076	+
384		269,281	-
1174		253,275	+
1294		195,648	+
1071		169,520	+
1071		169,520	+
1080		149,600	+
297		149,257	-
1254		134,583	+
1884		71,173	-
1409		20,283	-
1892		19,236	+
535		17,367	+
1364		15,271	-
541		15,132	+
156		12,947	+
1663		11,289	+
1615		10,245	-
865	Ovary	462,671,117	+
425		293,944,784	+
766		81,119,792	+
11	Ovary	37,500,953	+
859		438,572	+
559		329,307	+
1-48		327,029	+
2-18		292,580	+
2-62		283,251	+
2-29		275,814	+
1-05		164,614	+
916		103,501	+
1-31		12,005	-
240		883	+
4		859	-
195		251	-
896		212	-
193	Esophagus	960,314	+
149		856,547	+
973		672,893	+
1092		493,030	-
374		121,991	+
312		63,661	+
886	Liver	1,891,171	+
772		40,371	-
719	Pancreas	1,871,054	-
421		290,552	+
24	Healthy	18,634	-
184		17,584	-
76		15,209	-
134		10,491	+
322		8,166	-
131		7,943	-
69		1,321	-
185		181	-
218		137	-
96		49	-
