# Supplementary material for: Cancer Relevance of Circulating Antibodies Against LINE-1 Antigens in Humans
Source: Cancer Res Commun. 2023 Nov 8;3(11):2256–67. doi: 10.1158/2767-9764.CRC-23-0289 (PMC10631453; doi:10.1158/2767-9764.CRC-23-0289)
Supplement: Table S3 — Supplementary Table S3 shows IgG titers against ORF1p-derived peptides in healthy persons, lung, and ovarian cancer patients. [file crc-23-0289-s15.pdf]

Table S3. Titers of anti-ORF1p IgG against ORF1p-derived peptides in serum samples from healthy persons, lung, and ovarian cancer patients and healthy subjects (“Normal”).

	# Peptide																			
**# Sample**	**1**	**2**	**3**	**4**	**5**	**6**	**7**	**8**	**9**	**10**	**11**	**12**	**13**	**14**	**15**	**16**	**17**	**BSA**	**ORF1p titer**	**Cancer origin**
**865**	10,829	6,207	17,070	371	84	60	577	70	49	57	64	607	66	48	49	162	51	53	21,362,567,592	**Ovary**
**825**	17,075	12,340	1,916	62	53	237	177	59	45	74	73	456	79	54	54	6,724	60	39	776,796,582	**Ovary**
**766**	4,718	588	146	10,044	250	29	4,560	32	30	33	95	26	32	52	441	30	146	34	544,820,160	**Ovary**
**425**	24,803	15,878	28,485	1,370	128	121	2,165	129	117	114	96	1,834	130	93	101	478	73	93	293,944,784	**Ovary**
**11**	17,422	170	1,541	35	28	38	28	33	29	32	26	21	24	30	22	32	69	28	71,512,667	**Ovary**
**1206**	18,647	6,294	19,233	158	1,422	4,490	44	27	22	25	63	83	33	41	99	434	290	37	80,557,785	**Lung**
**1668**	17,063	17,745	13,915	163	6,630	675	94	94	63	82	87	160	101	145	111	117	1,471	107	15,484,421	**Lung**
**1390**	11,257	20,088	3,628	55	1,280	4,146	4,932	82	62	22	172	180	91	54	46	534	693	64	11,669,689	**Lung**
**373**	2,728	4,452	132	72	232	2,452	115	70	67	67	77	66	69	67	65	64	63	61	5,320,280	**Lung**
**1132**	3,146	8,326	38	85	66	47	359	81	29	36	128	135	60	75	115	50	90	59	1,483,048	**Lung**
**1376**	8,722	4,172	9,990	3,493	794	383	44	428	23	19	122	321	37	32	41	437	212	27	1,129,244	**Lung**
**1050**	1,318	169	7,744	20	24	23	35	26	20	22	165	284	63	47	51	23	52	35	1,103,537	**Lung**
**1189**	1,756	2,295	189	450	2,294	177	52	76	25	29	475	2,252	122	83	818	7,263	65	29	1,045,494	**Lung**
**1025**	4,252	65	9,884	58	79	66	60	39	137	27	2,344	2,187	256	189	600	828	659	70	469,697	**Lung**
**990**	506	2,950	38	46	46	83	40	53	38	40	303	335	270	73	44	38	93	45	369,144	**Lung**
**1174**	140	625	70	56	65	359	66	50	77	40	61	70	78	56	52	53	57	70	253,346	**Lung**
**1294**	519	1,021	55	29	35	33	34	34	52	44	253	133	65	63	55	132	45	53	195,660	**Lung**
**1071**	224	442	108	61	72	81	103	143	53	116	143	156	156	160	147	88	90	129	169,657	**Lung**
**468**	124	169	17,089	39	32	37	39	35	54	35	99	60	77	62	71	52	55	48	160,805	**Lung**
**1080**	298	45	5,528	40	41	45	41	40	34	37	454	319	68	77	47	35	45	51	149,610	**Lung**
**1577**	7,074	7,067	458	391	4,212	411	384	2,099	278	417	538	642	615	610	490	492	591	458	142,057	**Lung**
**1254**	1,273	2,042	70	39	26	26	28	30	29	22	47	174	78	42	40	31	908	45	134,588	**Lung**
**1410**	62	46	37	35	33	44	37	35	36	43	61	56	85	52	53	37	47	45	68,451	**Lung**
**1415**	1,954	93	84	64	63	66	73	135	66	82	97	106	179	111	129	91	399	85	53,857	**Lung**
**24**	9,571	334	1,791	184	214	195	263	287	199	234	333	419	346	972	303	281	554	236	18,634	**Normal**
**184**	1,484	689	737	601	756	588	792	1,175	542	802	1,230	1,092	1,079	1,317	940	854	914	861	17,584	**Normal**
**76**	745	127	8,736	125	136	130	153	154	108	138	164	238	208	217	163	185	231	144	15,209	**Normal**
**134**	177	1,960	52	54	50	54	52	61	47	57	75	271	50	62	57	45	96	58	10,491	**Normal**
**322**	93	74	2,360	61	52	126	57	94	56	59	243	97	1,362	69	66	56	176	63	8,166	**Normal**
**131**	2,893	1,055	8,179	795	857	778	917	1,024	720	874	984	1,503	1,202	1,324	1,089	922	992	828	7,943	**Normal**
**128**	411	57	6,071	45	103	73	52	284	36	54	49	88	143	73	62	39	161	71	6,825	**Normal**
**227**	400	253	684	257	329	286	327	370	246	339	547	473	408	520	365	471	302	338	5,711	**Normal**
**246**	131	55	1,561	51	64	56	60	54	54	52	62	75	701	89	73	67	85	80	5,480	**Normal**
**114**	1,414	533	951	724	711	613	549	613	480	488	544	921	783	982	909	527	488	429	2,655	**Normal**
**106**	1,801	63	63	51	46	48	72	7,897	42	57	112	172	1,431	228	93	63	7,960	65	2,092	**Normal**
**151**	404	92	86	67	76	86	91	83	62	80	117	1,521	152	991	122	85	100	128	1,655	**Normal**
