# Supplementary material for: Cancer Relevance of Circulating Antibodies Against LINE-1 Antigens in Humans
Source: Cancer Res Commun. 2023 Nov 8;3(11):2256–67. doi: 10.1158/2767-9764.CRC-23-0289 (PMC10631453; doi:10.1158/2767-9764.CRC-23-0289)
Supplement: Table S4 — Supplementary Table S4 shows the number of samples for five cancer types and distribution by disease stages. [file crc-23-0289-s16.pdf]

Table S4. Number of serum samples from patients with early (1-2) and advanced (3-4) disease stages.

Cancer type	Sample size		
	All samples	Stages 1-2	Stages 3-4
Ovary	N=979	N=193	N=786
Pancreas	N=124	N=40	N=84
Liver	N=217	N=67	N=150
Esophagus	N=377	N=79	N=298
Lung	N=907	N=90	N=817
