# Supplementary material for: Cancer Relevance of Circulating Antibodies Against LINE-1 Antigens in Humans
Source: Cancer Res Commun. 2023 Nov 8;3(11):2256–67. doi: 10.1158/2767-9764.CRC-23-0289 (PMC10631453; doi:10.1158/2767-9764.CRC-23-0289)
Supplement: Table S5 — Supplementary Table S5 shows the distribution of age between cancer and healthy subjects. [file crc-23-0289-s17.pdf]

Table S5. The age ranges of cancer patients and healthy subjects included in the analysis

Cancer type	N	Mean	SD	Min	Max
**Ovary**	N=979	60.26	12.96	18	95
**Pancreas**	N=124	63.57	10.41	38	85
**Liver**	N=217	64.69	10.29	23	89
**Esophagus**	N=377	64.82	10.33	36	91
**Lung**	N=907	66.21	9.76	36	91
**Healthy**	N=352	34.99	15.48	20	89
