# Supplementary material for: Cancer Relevance of Circulating Antibodies Against LINE-1 Antigens in Humans
Source: Cancer Res Commun. 2023 Nov 8;3(11):2256–67. doi: 10.1158/2767-9764.CRC-23-0289 (PMC10631453; doi:10.1158/2767-9764.CRC-23-0289)
Supplement: Table S6 — Supplementary Table S6 shows results of logistic regression analysis of anti-ORF1 IgG titers in cancer patients relative to healthy individuals after adjusting for age. [file crc-23-0289-s18.pdf]

Table S6. Logistic regression analysis of anti-ORF1 IgG titers in cancer patients relative to healthy individuals after adjusting for age

	Anti-ORF1p IgG titers before adjustment for age	Anti-ORF1p IgG titers after adjustment for age
**Odds ratio**	1.8	1.67
**95% confidence interval (CI)**	(1.56, 2.08)	(1.43, 1.95)
**p-value**	<0.0001	<0.0001
