# Supplementary material for: Cancer Relevance of Circulating Antibodies Against LINE-1 Antigens in Humans
Source: Cancer Res Commun. 2023 Nov 8;3(11):2256–67. doi: 10.1158/2767-9764.CRC-23-0289 (PMC10631453; doi:10.1158/2767-9764.CRC-23-0289)
Supplement: Table S7 — Supplementary Table S7 shows the results of linear regression analysis of association between ORF1p IgG titers and individual cancer types relative to healthy subjects after adjustment for age. [file crc-23-0289-s19.pdf]

Table S7. Linear regression analysis: association between ORF1p IgG titers and individual cancer types (all stages) relative to control after adjustment for age

Cancer Type	No adjustment		Adjusting for age	
	Difference (95% CI)	p-value	Difference (95% CI)	p-value
**Lung**	0.42 (0.31, 0.52)	<0.0001	0.33 (0.21, 0.44)	<0.0001
**Esophagus**	0.52 (0.39, 0.65)	<0.0001	0.44 (0.31, 0.57)	<0.0001
**Liver**	0.52 (0.37, 0.67)	<0.0001	0.44 (0.29, 0.6)	<0.0001
**Ovary**	0.29 (0.18, 0.4)	<0.0001	0.21 (0.1, 0.33)	0.0009
**Pancreas**	0.5 (0.32, 0.69)	<0.0001	0.43 (0.25, 0.61)	<0.0001
