# Supplementary material for: Cancer Relevance of Circulating Antibodies Against LINE-1 Antigens in Humans
Source: Cancer Res Commun. 2023 Nov 8;3(11):2256–67. doi: 10.1158/2767-9764.CRC-23-0289 (PMC10631453; doi:10.1158/2767-9764.CRC-23-0289)
Supplement: Table S8 — Supplementary Table S8 shows the results of linear regression analysis: association between ORF1p IgG titers and individual cancer types (stages 1-2) relative to healthy subjects after adjustment for age. [file crc-23-0289-s20.pdf]

Table S8. Linear regression analysis: association between ORF1p IgG titers and individual cancer types (stages 1-2) relative to control after adjustment for age

Cancer Type	No adjustment		Adjusting for age	
	Difference (95% CI)	p-value	Difference (95% CI)	p-value
**Lung**	0.24 (0.11, 0.36)	0.0016	0.11 (-0.04, 0.26)	0.4944
**Esophagus**	0.39 (0.2, 0.57)	0.0002	0.27 (0.07, 0.46)	0.0372
**Liver**	0.52 (0.32, 0.73)	<0.0001	0.42 (0.21, 0.63)	0.0006
**Ovary**	0.2 (0.06, 0.34)	0.0257	0.16 (0.01, 0.3)	0.1362
**Pancreas**	0.55 (0.3, 0.79)	<0.0001	0.44 (0.19, 0.68)	0.003
