# Supplementary material for: Cancer Relevance of Circulating Antibodies Against LINE-1 Antigens in Humans
Source: Cancer Res Commun. 2023 Nov 8;3(11):2256–67. doi: 10.1158/2767-9764.CRC-23-0289 (PMC10631453; doi:10.1158/2767-9764.CRC-23-0289)
Supplement: Table S9 — Supplementary Table S9 shows comparison of anti-ORF1p IgG titers among healthy subjects of different races. [file crc-23-0289-s21.pdf]

Table S9. Comparison of anti-ORF1p IgG titers among healthy individuals of the indicated races (Dunn's multiple comparison test with adjusted p-value)

Ethnicity (sample size)	Comparison	p-value
Black (N=37)	Black vs. Caucasian	p=0.9
Hispanic (N=143)	Hispanic vs. Black	p=0.8
Caucasian (N=167)	Caucasian vs. Hispanic	p=0.3
