# Supplementary material for: Cancer Relevance of Circulating Antibodies Against LINE-1 Antigens in Humans
Source: Cancer Res Commun. 2023 Nov 8;3(11):2256–67. doi: 10.1158/2767-9764.CRC-23-0289 (PMC10631453; doi:10.1158/2767-9764.CRC-23-0289)
Supplement: Table S10 — Supplementary Table S10 shows gender-specificity of anti-ORF1p IgG titers among healthy subjects and cancer patients. [file crc-23-0289-s22.pdf]

Table S10. Gender-specificity of anti-ORF1p IgG titers among healthy individuals and cancer patients. Mann-Whitney U-test, p-value < 0.05 is considered significant.

Cancer type	Sample size	p-value
**Healthy**	Male (N=159)	p=0.2
	Female (N=188)	
**Pancreas**	Male (N=52)	p=0.3
	Female (N=72)	
**Liver**	Male (N=185)	p=0.4
	Female (N=32)	
**Esophagus**	Male (N=320)	p=0.5
	Female (N=57)	
**Lung**	Male (N=451)	**p=0.004**
	Female (N=456)	
	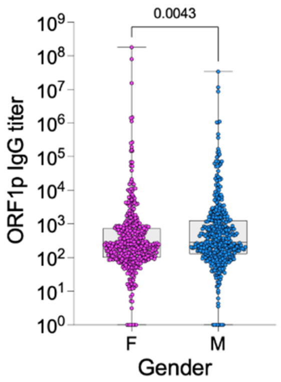	
