# Supplementary material for: Cancer Relevance of Circulating Antibodies Against LINE-1 Antigens in Humans
Source: Cancer Res Commun. 2023 Nov 8;3(11):2256–67. doi: 10.1158/2767-9764.CRC-23-0289 (PMC10631453; doi:10.1158/2767-9764.CRC-23-0289)
Supplement: Table S11 — Supplementary Table S11 shows comparison of anti-ORF1p IgG titers between indicated groups of subjects. [file crc-23-0289-s23.pdf]

Table S11. Anti-ORF1p IgG titers in subjects with myocardial infarction (MI) vs. healthy or chronic obstructive pulmonary disease/pneumonia (COPD/PN) patients vs. healthy and lung cancer patients. Mann-Whitney U-test, p-value < 0.05 is considered significant.

**Anti-ORF1p IgG titers**	**p-value**
COPD/PN patients (N=37) ~ healthy (n=304)	0.2
COPD/PN patients (N=37) **<** lung cancer patients (N=708)	0.0004
MI patients (N=33) ~ healthy (N=304)	0.9
